# Computationally repurposed drugs and natural products against RNA dependent RNA polymerase as potential COVID-19 therapies

**DOI:** 10.1186/s43556-021-00050-3

**Published:** 2021-09-20

**Authors:** Sakshi Piplani, Puneet Kumar Singh, David A. Winkler, Nikolai Petrovsky

**Affiliations:** 1 College of Medicine and Public Health, Flinders University, Bedford Park, 5046 Australia; 2 Vaxine Pty Ltd, 11 Walkley Avenue, Warradale, 5046 Australia; 3 School of Biochemistry and Genetics, La Trobe University, Bundoora, 3086 Australia; 4 Monash Institute of Pharmaceutical Sciences, Monash University, Parkville, 3052 Australia; 5 School of Pharmacy, University of Nottingham, Nottingham, NG7 2RD UK

**Keywords:** RNA-dependent RNA polymerase, SARS-CoV-2, Drug repurposing, Molecular dynamics, Molecular docking

## Abstract

Repurposing of existing drugs and drug candidates is an ideal approach to identify new potential therapies for SARS-CoV-2 that can be tested without delay in human trials of infected patients. Here we applied a virtual screening approach using Autodock Vina and molecular dynamics simulation in tandem to calculate binding energies for repurposed drugs against the SARS-CoV-2 RNA-dependent RNA polymerase (RdRp). We thereby identified 80 promising compounds with potential activity against SARS-Cov2, consisting of a mixture of antiviral drugs, natural products and drugs with diverse modes of action. A substantial proportion of the top 80 compounds identified in this study had been shown by others to have SARS-CoV-2 antiviral effects in vitro or in vivo, thereby validating our approach. Amongst our top hits not previously reported to have SARS-CoV-2 activity, were eribulin, a macrocyclic ketone analogue of the marine compound halichondrin B and an anticancer drug, the AXL receptor tyrosine kinase inhibitor bemcentinib. Our top hits from our RdRp drug screen may not only have utility in treating COVID-19 but may provide a useful starting point for therapeutics against other coronaviruses. Hence, our modelling approach successfully identified multiple drugs with potential activity against SARS-CoV-2 RdRp.

**Supplementary Information:**

The online version contains supplementary material available at 10.1186/s43556-021-00050-3.

## Introduction

SARS-CoV-2 (severe acute respiratory syndrome coronavirus 2) is the virus responsible for the COVID-19 pandemic that first appeared in 2019 and has caused major morbidity and mortality worldwide. At the end of May 2021, there had been more than 170 million reported cases and 3.5 million deaths due to COVID-19. The world has faced unprecedented challenges in managing COVID-19, triggering a major effort to find vaccines and drugs effective against SARS-CoV-2. Given the immediacy of the problem, the fastest approach is to identify existing drugs or natural products that could be quickly repurposed for treatment of COVID-19. Such agents can be rapidly deployed into human trials as their safety and pharmacokinetics in man are already well known.

Computational methods offer considerable promise for rapid screening for potential drugs against SARS-CoV-2 protein targets. For example, recent work demonstrated the ability to undertake computational de novo drug design based on the recently identified structure of M^pro^, the main SARS-CoV-2 protease [1]. Another interesting but less studied target for SARS-CoV-2 drug development is its RNA-dependent RNA polymerase (RdRp). Zhu et al. recently reviewed the biochemical properties of RdRp and described cell-based assays suitable for high-throughput screening of RdRp drug candidates [2]. RdRp plays a crucial role in the SARS-CoV-2 replicative cycle with its active site being highly conserved and accessible, making it an ideal drug target. Furthermore, all DNA and RNA viruses employ RdRp proteins for replication and transcription of viral genes, suggesting that similar computational techniques could be broadly applicable to find drugs against other viruses [2]. RdRps share several sequence motifs and tertiary structures between all RNA virus types: positive-sense RNA; negative-sense RNA; and dsRNA. The core structure of RdRp resembles a right-hand, with palm, thumb, and finger domains. Five of the seven classical catalytic RdRp motifs (A – E) are in the most conserved palm domain, while the remaining two (F and G) are in the finger domains. The structurally conserved RdRp core and related motifs are important for the catalytic role of viral RdRp and thus represent potential targets for drug intervention. While the criteria for substrates differ, all known RdRps share the same catalytic mechanism. After host cell infection viral RdRp participates in formation of the molecular machinery for genome replication by complexing with other transcription factors. It initiates and regulates the elongation of the RNA strand, which involves the addition of hundreds to thousands of nucleotides. When incorporated into the newly synthesised RNA chain, nucleotide analogues, such as remdesivir, will block the RNA elongation catalysed by RdRp.

Computational methods can quickly identify drugs for repurposing in pandemics where speed is of utmost importance. There are two main ways of using computational methods to predict the activities of drugs – ligand-based and structure-based. Ligand-based methods use statistical and machine learning methods to generate mathematical relationships between the drug structures and their biological activity and are trained on large experimental data sets. The paucity of experimental data on drugs active against RdRp make ligand-based methods impractical, leaving structure-based methods as the only viable computational approaches. Structure-based methods use a 3D structure of the target protein (from x-ray, nuclear magnetic resonance (NMR) or in silico homology modelling) together with computational docking methods to estimate the energy of interactions of ligand molecules with the protein binding site. The compounds with the most favourable interactions (docking scores) are then subjected to molecular dynamics (MD) simulations to calculate more accurate binding poses and binding energies.

**Fig. 1:**
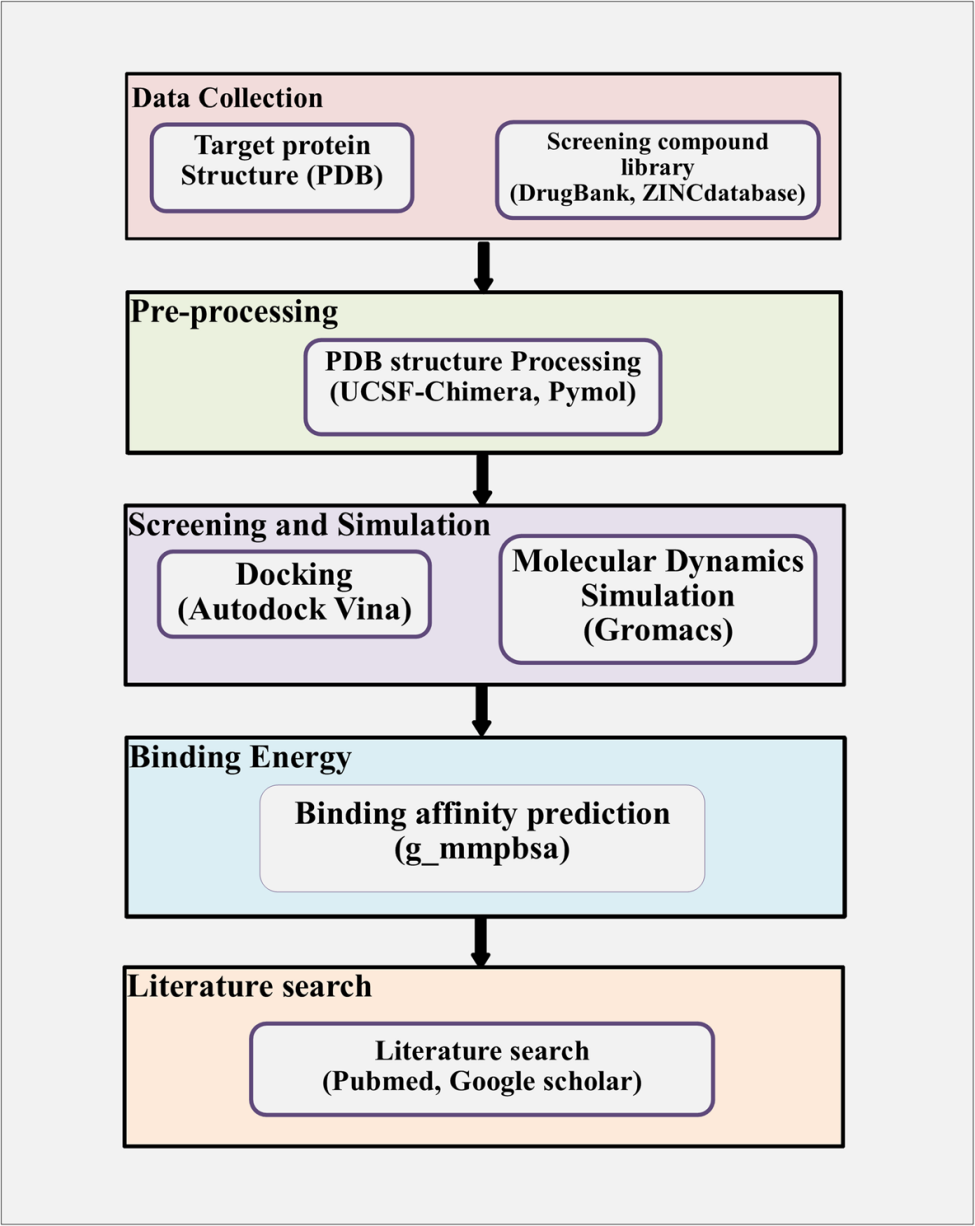
Computational workflow for in silico screening of existing drugs and natural compounds for potential activity against SARS-Cov-2 RdRp protein

Here we show how our in silico drug discovery pipeline (Fig. 1) consisting of molecular docking followed by high-throughput molecular dynamics (MD) simulations, can screen a large number of existing drugs to generate a candidate list of RdRp inhibitors. Subsequently, MD calculations were used to predict the binding energies and optimal binding poses for the 80 best scoring RdRp hits. We describe the properties of the top candidates based on binding affinity and novelty. The paper is organized as follows: the Introduction outlines the rationale for selection of RdRp and the selected screening methodology; the Results section lists the identified repurposed drugs with predicted highest affinity for RdRp and provides details on the molecular interactions between key drug candidates and the active site of RdRp; the Discussion compares the findings with relevant experimental research from the literature; and the Materials and Method section provides complete details on how the computational studies were conducted to allow our results to be replicated.

## Results

### RdRp ligand docking followed by molecular dynamics simulations

Many computational studies have attempted to predict which existing drugs may inhibit the SARS-CoV-2 main protease, M^pro^, but far fewer studies have targeted the viral polymerase, RdRp. The computational workflow used for estimating the binding affinities of candidate drugs for the RdRp binding pocket is shown in Fig. 1. Substantial improvements in protein-ligand docking results can be achieved through MD simulation [3]. Hence we used Vina docking with MD simulation to yield more accurate results over docking alone. The RdRp binding free energies for the top 80 hits (Supplementary Table S1) as calculated by either of two methods (see Methods section) correlated very well (r^2^ = 0.84), and the free energies calculated by the thermodynamic cycle correlated with the Vina docking scores (r^2^ = 0.64). The large RdRp binding pocket (area 2920 Å^2^ and volume 5335 Å^3^) prefers larger ligands, many of which are quite flexible. Binding energy penalties due to ligand entropy were likely to be significant. Hence, substantial correlation between the Vina scores and the binding energies from MMPBSA and thermodynamic cycle was important because these algorithms treat ligand entropy approximately and in different ways [4].

The 20 drugs with the highest predicted binding to the RdRp active site together with their molecular mechanics Poisson-Boltzmann surface area (MMPBSA), a method to estimate interaction free energies, and thermodynamic cycle binding energies are summarized in Table 1. Most of the drugs in the top 20 had relatively large complex structures and substantial ligand flexibility. Antiviral drugs accounted for half of the top 20 list with their structures and plots showing the interactions of the drugs with residues in the active site of RdRp (ligplots) are shown in Supplementary Fig. S1. The remaining compounds in the top 20 hits included many natural products or their derivatives; their ligplots are summarized in Supplementary Fig. S2).

**Table 1:**
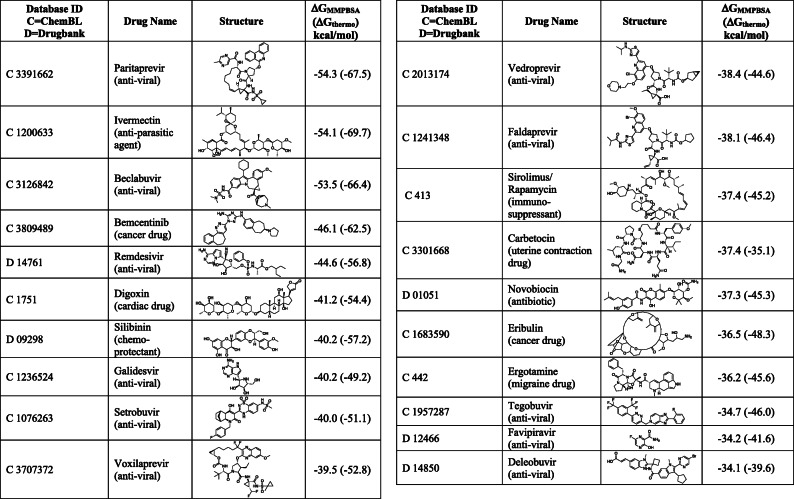
Top 20 ranked SARS-CoV-2 RdRp-active drugs based on binding energy by MMPBSA score

### Correlations of model predictions with in vitro measurements of virus activity

**Fig. 2:**
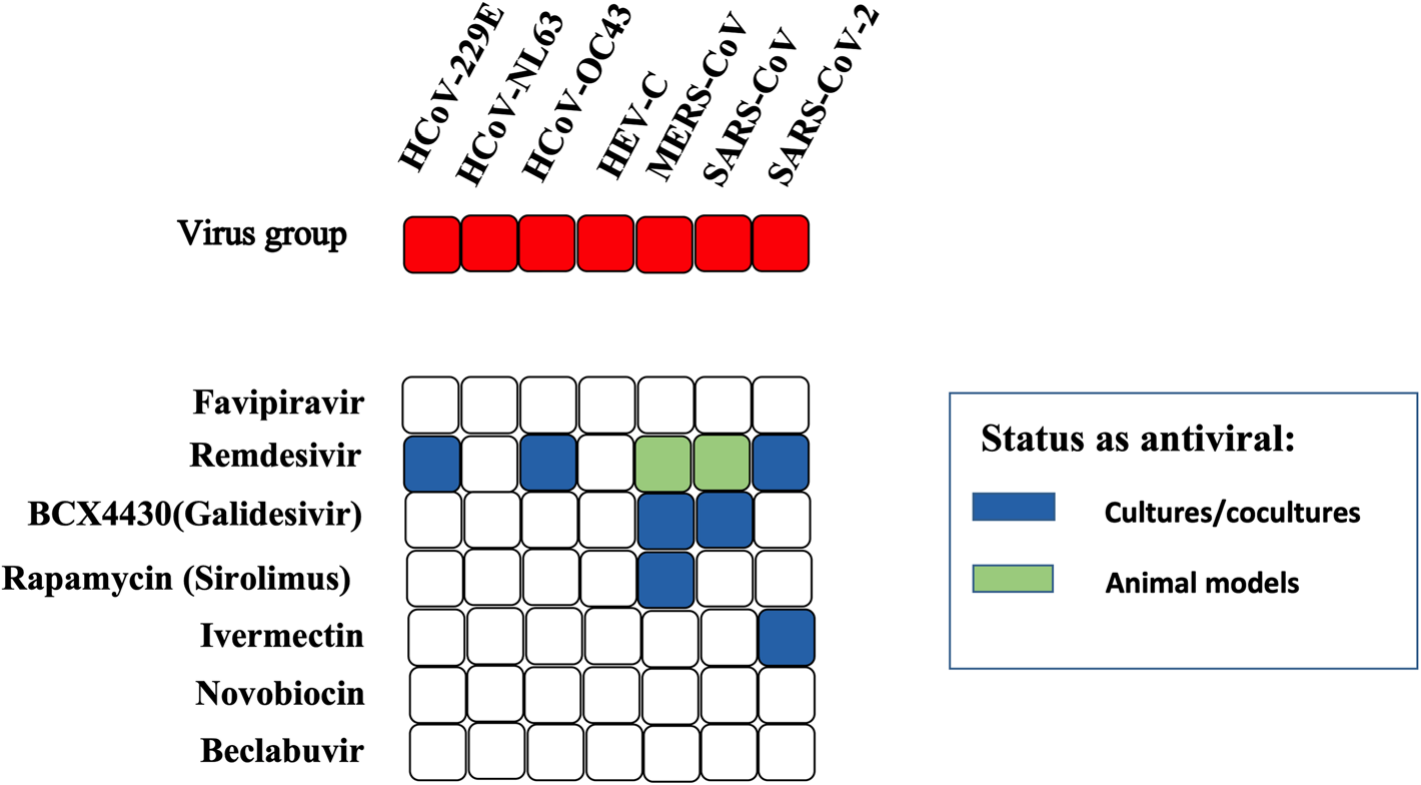
Anti-coronavirus activity. Results of a search at www.drugvirus.info. A colour code is used to indicate the level of evidence of anti-viral activity for a particular drug. The relevant viruses searched are shown in the red bar at the top of the figure

Many of the top antiviral drug hits had already been experimentally assessed for activity against SARS-CoV-2 and other coronaviruses such as SARS and MERS CoV. The antiviral drug database at www.drugvirus.info allows users to select antiviral drugs and particular viruses and then generates a matrix table detailing which drugs have been shown to be active against which of the selected viruses. A search of our top hits against this database confirmed several had known -coronavirus activity, including some against SARS-CoV-2 (Fig. 2). Remdesivir showed the broadest activity having been shown in cell cultures to be active against HCoV-229E, HCoV-OC43, MERS, and SARS as well as SARS-CoV-2. When we performed a more extensive search of the literature using PubMed and Google Scholar, we identified more than 100 studies relating to our top 80 hits, with descriptions of in vitro and/or in vivo data supporting activity of these drugs against SARS CoV-2 (Supplementary Table S1). Notably, the calculated binding energy of the top antiviral drugs we identified as RdRp targets, e.g., paritaprevir and beclabuvir, were very similar to published binding affinity data. This provides validation that our computational methods yielded results comparable with other published studies.

### Comparing our model with other docking studies of known antiviral drugs

We sought to compare the data generated by our model with other RdRp docking study results. Beg and Athar used AutoDock Vina with a homology modelled structure for RdRp and similarly found paritaprevir, beclabuvir, and favipiravir to have high docking scores [5]. Cozac et al. used a combined docking and machine learning approach to identify inhibitors of RdRp polymerases from HCV, poliovirus, dengue virus, and influenza virus. They identified faldaprevir, vedroprevir, beclabuvir and remdesivir as having good docking scores with predicted RdRp IC_50_/EC_50_ values below 5 μM [6]. Dutta et al. used a homology model to identify antiviral drugs against COVID-19 and predicted beclabuvir to have an IC_50_ of 50 nM, with many antiviral agents having docking scores higher than remdesivir (− 7.4 kcal/mol) or radalbuvir (− 7.4 kcal/mol), e.g. beclabuvir (− 10 kcal/mol), tegobuvir (− 9.7 kcal/mol), dasabuvir (− 9.4 kcal/mol), lomibuvir (− 11 kcal/mol), setrobuvir (− 10.5 kcal/mol) [7]. Unlike our approach, none of these studies used MD refinement of docked compounds to improve the accuracy of the binding free energy predictions. Notably, remdesivir was predicted to be active against SARS-CoV-2 RdRp in several studies but its in vitro activity is low (IC_50_ 3.7 μM in Vero cells), consistent with recent clinical studies showing only limited efficacy in treating COVID-19 infection in man [8–10]. This is in accord with our study results which predicted remdesivir to have relatively low RdRp binding. Elfiki used AutoDock Vina docking and MD simulations (50 ns runs) to model RdRp and predicted antiviral drugs sofosbuvir, ribavirin, galidesivir, remdesivir, favipiravir, cefuroxime, tenofovir, setrobuvir, and hydroxychloroquine to bind to RdRp [11]. Ahmed et al. used docking and 100 ns MD simulations on 76 antiviral drugs, predicting remdesivir, raltegravir, and simeprevir as having the best binding free energies, ranging from − 32 to − 38 kcal/mol [12]. Aouldate et al., undertook virtual screening of 50,000 chemical compounds from the CAS Antiviral COVID19 database to identify one compound (833463–19-7) predicted to bind well to RdRp [13]. Banerjee et al. used protein modelling and computational docking to investigate the effects of common mutations in RdRp, 3CLpro, and PLpro sequences and identified two RdRp mutations in the Indian population with prevalence > 5% with docking using Autodock Vina and predicted elbasvir followed by remdesivir and methylprednisolone as the most active against Indian RdRp mutants [14]. Overall, the above studies used less rigorous methods than ours and more limited drug libraries and chiefly identified already known antiviral compounds such as remdesivir. Notably, the antiviral drugs in our own top 80 RdRp hits had a high degree of overlap with the more limited hits of these other in silico antiviral drug studies.

### Identification of natural products as potential RdRp drug candidates

**Fig. 3:**
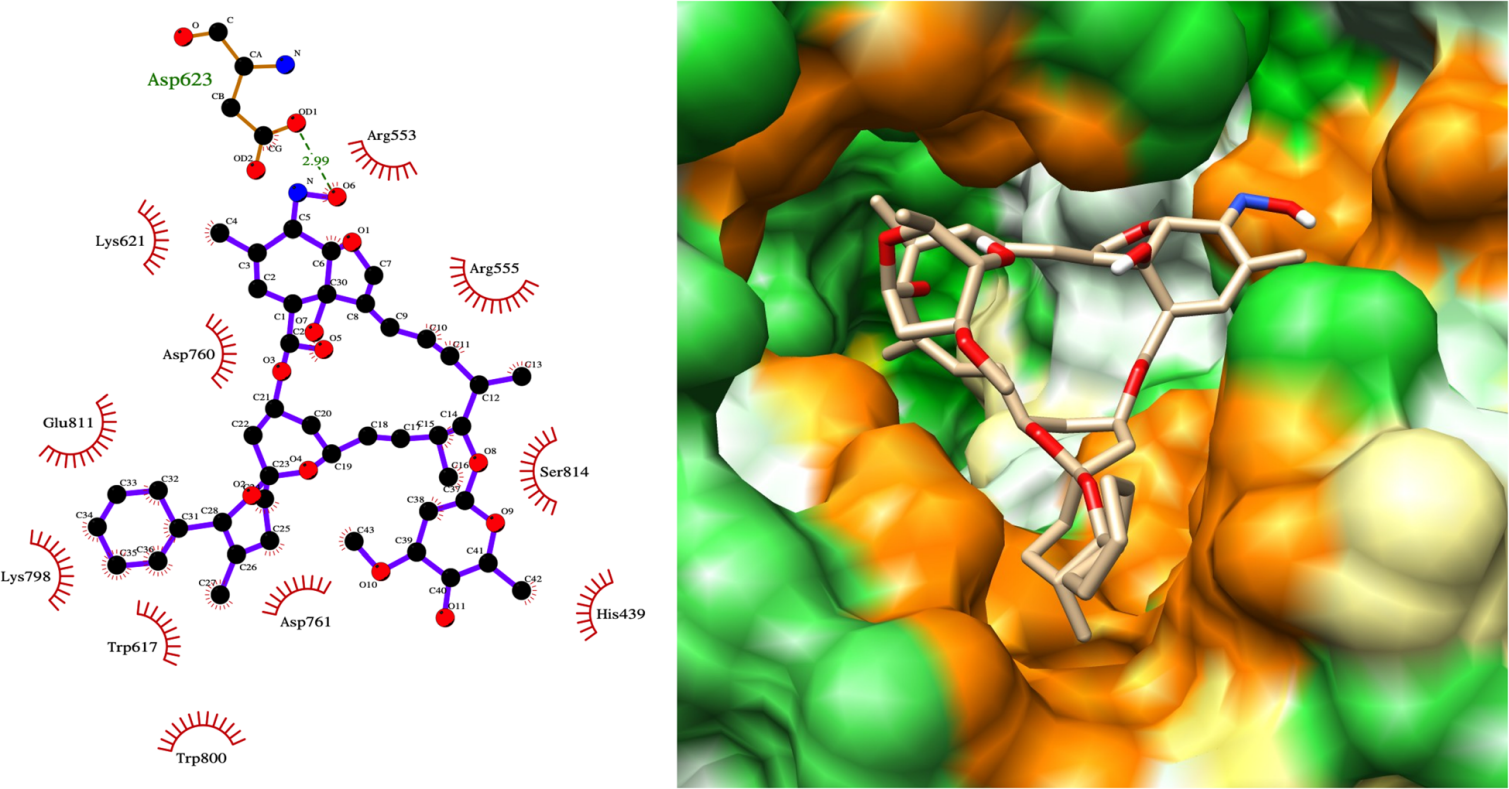
LigPlot and hydrophobic protein surface representation of the main interactions between ivermectin and RdRP

Amongst drugs for COVID-19 repurposing, the natural products in our top 20 hits were of high interest given their relative novelty and molecular diversity. The drug we identified as having the second highest binding affinity to RdRp (after paritaprevir) was the natural product, ivermectin. Invermectin fitted snugly into the RdRp binding pocket forming a hydrogen bond with Asp623 of RdRp (Fig. 3). Ivermectin and other avermectins and milbemycins are broad spectrum antiparasitic macrocyclic lactones derived from the bacterium *Streptomyces avermitilis*. Ivermectin’s anti-parasitic mode of action is to enhance inhibitory neurotransmission by binding to glutamate-gated chloride channels. It has been shown to be effective against several positive-sense single-strand RNA viruses including SARS-CoV-2, and has been proposed as a strong COVID-19 drug candidate [15–17]. It inhibits replication of SARS-CoV-2 in monkey kidney cell culture with an IC_50_ of 2.2–2.8 μM [9, 15]. Similarly, Janabi et al. predicted favourable binding energies of ivermectin and several milbemycins for RdRp using AutoDock Vina, without subsequent simulation of the protein-ligand complexes [18]. Several trials of ivermectin in COVID-19 are already in progress (www.ClinicalTrials.gov -NCT04390022, NCT04602507).

**Fig. 4:**
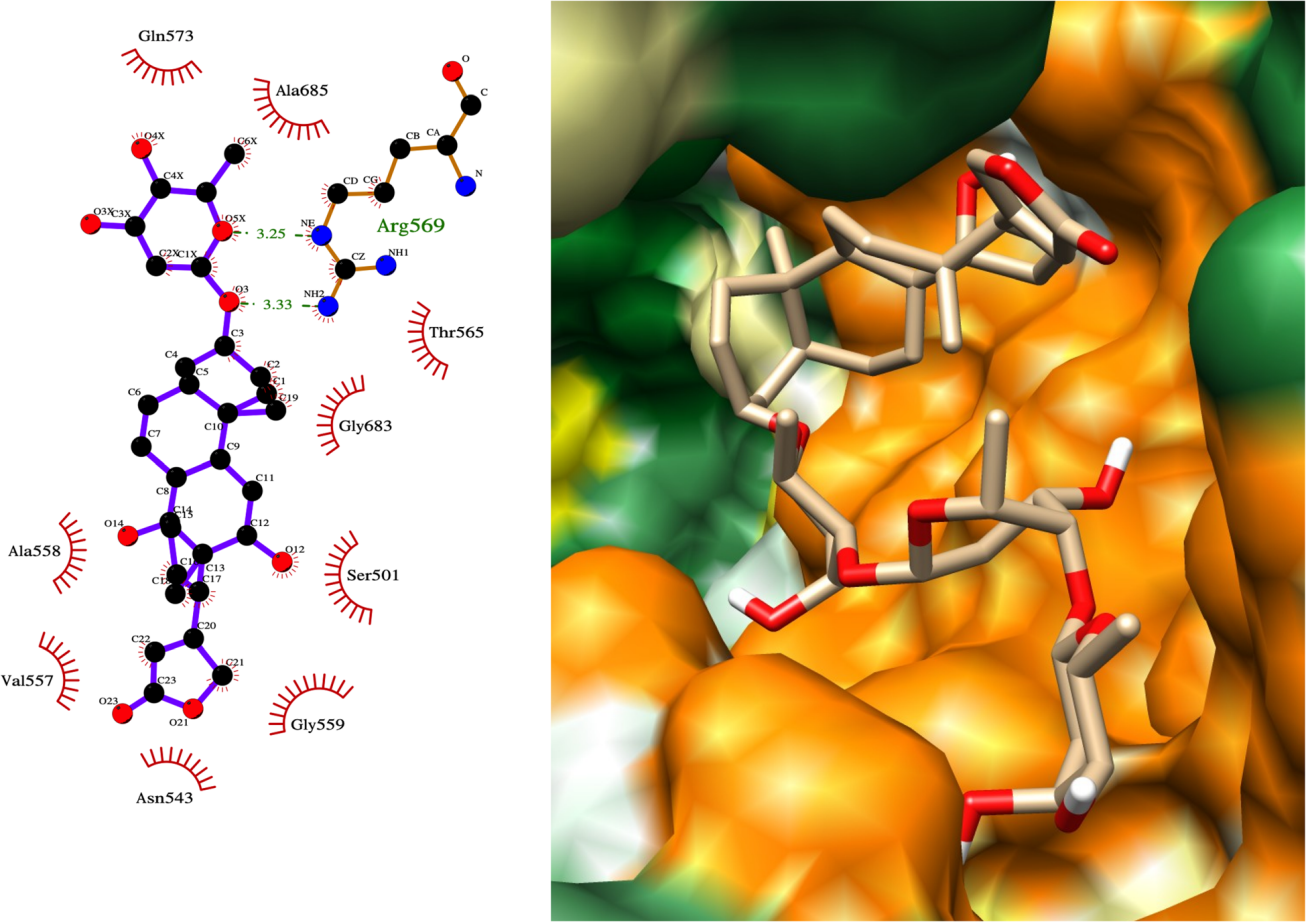
LigPlot and hydrophobic protein surface representation of the main interactions between digoxin and RdRP

Another natural product we predicted to have high activity against RdRp was digoxin. Digoxin is a widely used cardiac drug used to treat heart arrythmias and cardiac failure and has a large complex structure that fitted well into the RdRp binding pocket, forming multiple hydrogen bonds with Ser682, Arg553, Arg624 and Cys622. (Fig. 4). Interestingly, digoxin was also predicted to inhibit the interaction of SARS-CoV-2 with ACE2 by Kalhor et al. [19]. No previous studies have reported potential inhibition of RdRp by digoxin. Very recently, digoxin was shown to have potent in vitro antiviral effects against SARS-CoV-2 in Vero cells (IC_50_ 37 nM) by Cho et al. and also by Jeon et al. (IC_50_ of 190 nM) [20, 21]. Notably, the activity of digoxin against SARS-CoV-2 in these cellular assays was substantially higher than seen with chloroquine or remdesivir.

**Fig. 5:**
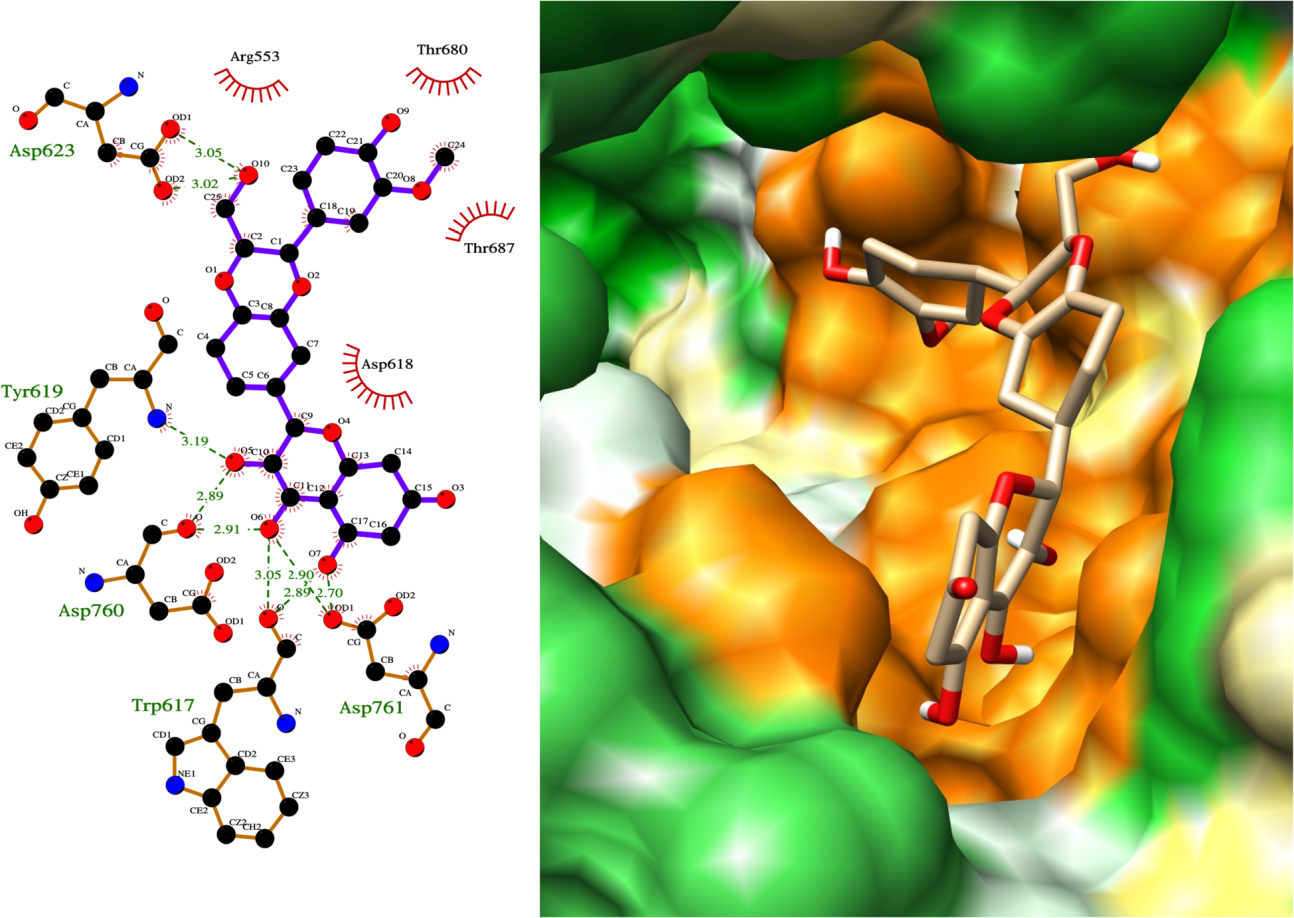
LigPlot and hydrophobic protein surface representation of the main interactions between silibinin and RdRP

Another natural product predicted to have high binding for RdRp was silibinin, which formed multiple hydrogen bonds to Asp623, Tyr619, Asp760, Trp617 and Asp761 (Fig. 5). Silibinin is a flavonolignan that is the major active constituent of silymarin, a standardized extract of the milk thistle seeds, with potential roles as an antioxidant, antineoplastic drug, and hepatoprotectant. Silibinin was also predicted to be a potential inhibitor of RdRp by two other computational docking studies and is the subject of planned clinical trials [22].

**Fig. 6:**
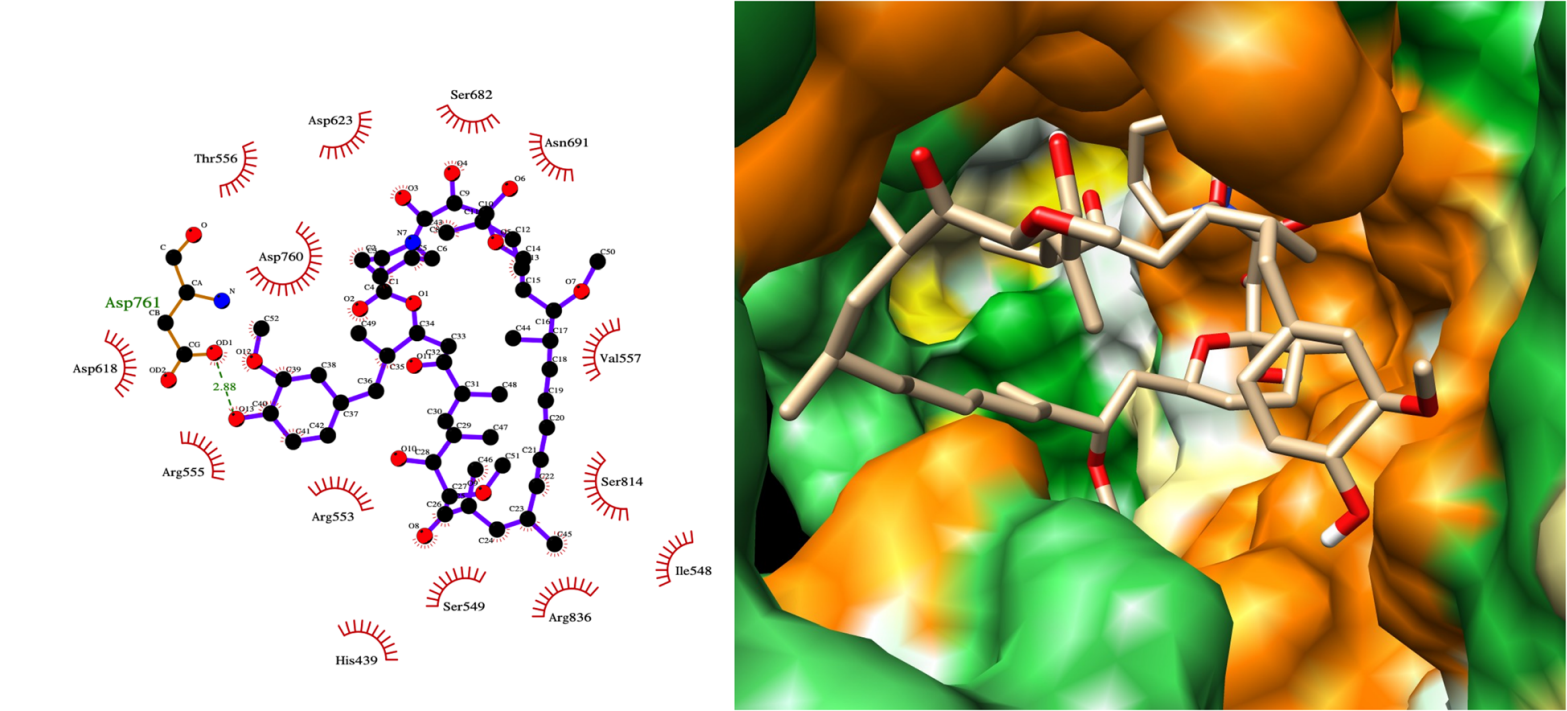
LigPlot and hydrophobic protein surface representation of the main interactions between rapamycin and RdRP

Rapamycin (sirolimus) was another natural product we predict to have high activity against RdRp, forming a hydrogen bond with Asp761 (Fig. 6). Rapamycin was also identified as an RdRp target by another modelling study [23]. Rapamycin is a macrolide antifungal metabolite that has immunosuppressant activity and is used to prevent organ transplant rejection. Rapamycin is in COVID-19 clinical trials (https://clinicaltrials.gov/ct2/show/NCT04461340).

Carbetocin was also in our top 20 hits (Supplementary Fig. S2). Carbetocin is a synthetic analogue of the natural product oxytocin in which the labile disulfide bond in the macrocycle is replaced by a thioether. Carbetocin was predicted to be in the top 10 RdRp inhibitors by Ahmad et al. [24]. It is an approved drug for uterine contraction and control of postpartum bleeding, and it has a transcriptomic signature suggestive of anti-inflammatory and immune stimulatory effects [25].

Eribulin, a fully synthetic macrocyclic ketone analogue of the marine natural product halichondrin B and potent anti-mitotic anticancer agent was another of our top 20 hits, forming 4 hydrogen bonds with RdRp (Supplementary Fig. S2). Although eribulin is active against other viral RdRps, there are no previous reports of it having activity against SARS-CoV-2 RdRp [26]. Hence eribulin might be an interesting candidate to be screened for activity against SARS-CoV-2.

Novobiocin, an aminocoumarin antibiotic that is produced by the actinomycete *Streptomyces niveus*, was also identified in our RdRp screen. Novobiocin targets DNA gyrase, a bacterial type IIA topoisomerase. It was identified by another study as a high binder to RdRp using MoleGro virtual docker software [27].

Our screen also identified ergotamine as a potential natural product RdRp inhibitor. Ergotamine and related ergot alkaloids were predicted by others to bind several SARS-CoV-2 molecular targets, including the main protease, M^pro^. There are literature reports of ergotamine binding to SARS-CoV-2 RdRp and another in silico study predicted ergotamine had an IC_50_ of 190 μM for RdRp [28, 29].

### Other RdRp drug candidates of interest

Our screen also identified bemcentinib as a potential RdRp binder (Supplementary Fig. S2). Bemcentinib selectively inhibits AXL receptor tyrosine kinase activity and is under development as an anti-cancer drug. COVID-19 has been shown to use AXL to enter into some cells, with bemcentinib profoundly inhibiting virus entry into those cells [30]. Bemcentinib exhibits in vitro activity against SARS-CoV-2 with Liu et al. reporting 10–40% inhibition at 50 μM in Vero cells [31]. It exhibited an IC_50_ of 100 nM and CC_50_ of 4.7 μM in human Huh7.5 cells and an IC_50_ was 470 nM and CC_50_ of 1.6 μM in Vero cells [32]. It is currently undergoing trials for COVID-19 treatment.

## Discussion

Overall, the top 80 RdRp hits predicted by our docking studies were strongly populated by particular classes of drugs, notably antivirals but also kinase inhibitors (e.g. imatinib, ponatinib, rebastinib, lonafarnib, tivantinib, entrectinib), antibiotics (e.g novobiocin, quinupristin, dalfopristin, rifapentine, erythromycin, itraconazole, bafilomycin A1) and anti-cancer drugs (e.g. eribulin, etoposide, quarfloxin, epirubicin, brequinar, idarubicin, midostaurin). Other diverse drugs in the top 80 hits included eltrombopag (thrombocytopenia drug), dutasteride (prostrate drug), telmisartan (anti-hypertensive), conivaptan (hyponatremia drug), sertindole (antipsychotic), vapreotide (somatostatin analogue) and bromocriptine (Parkinson’s disease drug). Although we were not able to perform wet-lab validation of our hits, we demonstrated from a search of the literature that a substantial percentage (> 30%) of our hits had been shown experimentally to inhibit RdRp and/or to have in vitro SARS-CoV-2 antivirus activity (Supplementary Table S1). For example, indinavir had been shown to inhibit SARS-CoV-2 with an EC_50_ > 10 μM and CC_50_ > 50 μM in A549-hACE2 cells and EC_50_ of 59 μM and CC_50_ > 81 μM in Vero cells [33, 34]. Another drug we predicted, eltrombopag, had been shown to inhibit SARS-CoV-2 with an IC_50_ of 8 μM and CC_50_ > 50 μM in Vero cells and an IC_50_ of 8 μM Calu-3 cells [21, 35]. Yet another drug we predicted, elbasvir, had been shown to inhibit SARS-CoV-2 with an EC_50_ of 23 μM in Huh7-hACE2 cells [36]. Similarly, ponatinib was active against SARS-CoV-2 in HEK-293 T cells with an EC_50_ of 1 μM and a CC_50_ of 9 μM and grazoprevir had an EC_50_ of 16 μM and CC_50_ of > 100 μM in Vero E6 cells [36]. Itraconazole had an EC_50_ of 2.3 μM in human Caco-2 cells and an IC_50_ against M^pro^ of 110 μM [37, 38]. Ciclesonide had an EC_90_ of 5 μM in Vero cells and EC_90_ of 0.55 μM in differentiated human bronchial tracheal epithelial cells, blocking viral RNA replication by > 90% and currently being in trials in COVID-19 patients [21, 39]. Brequinar had an in vitro EC_50_ of 0.3 μM and CC_50_ > 50 μM in Vero E6 cells [40]. Telmisartan, an antihypertensive drug we predicted to inhibit RdRp, is already in COVID-19 clinical trials (NCT04356495, [41]). This thereby provided independent validation of the ability of our RdRp modelling approach to identify drugs active against SARS-CoV-2.

Interestingly, several of our RdRp hits have been shown to inhibit other SARS-CoV-2 protein targets. Simeprevir, one of the drugs we predicted to inhibit RdRp, was also shown to inhibit M^pro^ with an IC_50_ of 10 μM and reduced SARS-CoV-2 infectivity in vitro with an EC_50_ of 4 μM and CC_50_ 19 μM in Vero cells [42]. Imatinib, was shown to reduce M^pro^ activity at 10 μM with an EC_50_ of 8 μM in A549 cells and inhibited SARS-CoV-2 with an IC_50_ of 3-5 μM and CC_50_ > 30 μM [43, 44]. Conivaptan, was also shown to inhibit M^pro^ with an IC_50_ of ~ 10 μM in 293 T cells and inhibited SARS-COV-2 with IC_50_ of 10 μM in Vero cells and 4 μM in ACE2-A549 cells [43, 45]. The fact that a considerable number of our RdRp hits have also been shown to inhibit M^pro^ suggests that the active site of the RdRp polymerase and M^pro^ protease likely share common features facilitating their inhibition by the same drugs. Drugs that suppress a virus through multiple different mechanisms could be advantageous, as this makes it harder for the virus to mutate and become drug resistant, as to be successful it would need to escape both antiviral mechanisms.

In addition to many of our top 80 hits having had in vitro activity shown, several have already progressed to clinical trials. Indeed, some of our top 80 hits could have other beneficial effects for COVID-19 treatment given their primary drug actions. For example, in addition to a direct antiviral effect, rapamycin might usefully suppress inflammation and cytokine storms seen in COVID-19.

In conclusion, our virtual screening approach used molecular docking and MD simulations to calculate binding energies to identify 80 compounds with potential activity against SARS-CoV-2 RdRp. The top hits comprised antiviral agents, antibiotics, kinase inhibitors, natural products and other drugs with diverse modes of action. Strong support for our hits comes from published studies that confirm that many of our hits have promising activity against SARS-CoV-2.

## Materials and methods

### Protein structure preparation and grid preparation

The crystal structure of the SARS-CoV-2 RdRp was downloaded from RCSB PDB (https://www.rcsb.org/structure/6M71) with a reported resolution of 2.90 Å. UCSF Chimera package (https://www.cgl.ucsf.edu/chimera/) was used to prepare the protein by adding hydrogen atoms and missing residues and loops and removing non-essential and non-bridging water molecules [46]. Autodock Vina requires a specific file format and the assigning of hydrogen polarities and Gasteiger charges to protein structures and conversion of protein structures from the .pdb file format to .pdbqt format. This was carried out automatically using the AutoDock Tools (ADT) software [47].

### Screening databases

A total of 8773 drugs were downloaded from the Drugbank database and 13,308 from the CHEMBL (FDA approved) database. The drugs were converted from .sdf format to .pdbqt format by Raccoon [47].

### Docking methodology

We docked the small molecules from the drug database against the RdRp protein structure using the widely-used and robust AutoDock Vina (version 1.1.3) package [47]. This algorithm uses gradient-based conformational searches and an empirical scoring function based on energies of interaction between the ligand and receptor. The method is very flexible, extensively validated with different proteins and ligands, easily scripted, and is deployable on large multi-CPU or -GPU machines. Vina can successfully dock extremely large, small molecule drug libraries across a range of proteins with different physicochemical properties, to discover new potent drug leads. Vina is part of the Autodock package that includes scripts for generating specific file formats required for docking calculations and for establishing computational grids around each protein automatically. It requires the atom types in ligands and proteins to be set correctly, all hydrogens to be removed except polar hydrogens, and calculation of atomic partial charges required by Vina. Vina generates a grid around and through each protein and calculates energies of interaction of probe atoms at each grid position, including those inside protein pockets. We used a grid resolution of 1 Å, a maximum of 10 binding modes to be docked, and an exhaustiveness level of 8. This specifies the number of independent runs performed. The genetic algorithm option was employed to optimize ligand binding conformations in the RdRP active site. Thus, repurposing candidates were each docked into the active site of SARS-CoV-2 RdRP (refcode 6 M71). The bash script vina_screen.sh (Supplementary Information) was used to calculate the grid centre and size. A python script script1.py (Supplementary Information) identified compounds with the strongest binding interactions with the RdRP active site. These were subsequently subjected to molecular dynamics simulations to improve their docking poses and calculated binding energies. UCSF Chimera was used to analyse the docked structures and the hydrogen-bond and hydrophobic interactions were plotted using LigPlot+ software [46, 48]. Fifty compounds with the best docking scores were selected from each of the Drugbank and ChEMBL lists. As 20 compounds were common to both databases, the list of highest scoring repurposing candidates contained 80 molecules that were subsequently subject to MD simulations.

### Molecular dynamics simulation

The complexes between RdRp and each of the 80 candidate drugs were first minimized using CHARMm force field and used as starting geometries for MD simulations.. We used Swissparam (http://www.swissparam.ch/) to generate topology files for ligands [49]. MD simulations used gromacs2020 GPU-accelerated version (http://www.gromacs.org/) and the periodic boundary conditions from the CHARMm force field I in the ORACLE server [50]. For the MD simulations of the docked complexes, a truncated octahedral box of TIP3P water molecules was used to solvate the complex. Na + or Cl − counter ions were added to neutralize charges by the tleap program. The Particle Mesh Ewald (PME) method with a van der Waals (VdW) interaction cut-off distance of 12.0 Å was used to calculate long-range electrostatic interactions. The whole protein-ligand systems were simulated without restraints. Two thousand five hundred cycles of steepest descent minimization were applied followed by 5000 cycles of conjugate gradient minimization. MD simulations heated each system from 0 to 300 K, in the NVT ensemble, for 50 ps. A Langevin thermostat with a force constant of 2.0 kcal/mol·Å^2^ and a coupling coefficient of 1.0/ps were applied to the complex. Subsequently, a 20 ns MD simulation production run was performed with appropriate periodic boundary conditions for each system in the NPT ensemble at a constant temperature of 300 K. The SHAKE algorithm was utilised during the MD simulations to constrain all covalent bonds involving hydrogen atoms. All simulations used a time step of 2 fs. Structural stability was monitored by root-mean-square deviation (RMSD) and RMSF values for the Cα atoms of the protein. We ran MD simulations for up to 100 ns on several compounds to make sure 20 ns was sufficient for convergence. Three replicate runs starting from different random seeds were used to estimate uncertainties in binding energy.

We calculated the binding free energies of the protein-ligand complexes by two methods. The energies of solvated small molecule ligands, the SARS-CoV-2 RdRp protein, and the bound complex were used to calculate the binding energy by subtraction.

1 $$ \Delta \mathrm{G}\ \left(\mathrm{binding},\mathrm{aq}\right)=\Delta \mathrm{G}\ \left(\mathrm{complex},\mathrm{aq}\right)-\left(\Delta \mathrm{G}\ \left(\mathrm{protein},\mathrm{aq}\right)+\Delta \mathrm{G}\ \left(\mathrm{ligand},\mathrm{aq}\right)\right). $$

The MMPBSA tool in gromacs2020 was used to calculate binding energies form the nonbonded interaction energies of the complex. This was performed using the GMXPBSA 2.1 package that uses Bash/Perl scripts to streamline MMPBSA calculations of the structural ensembles generated from gromacs trajectories [51]. It also automatically calculates binding free energies for protein–protein or ligand–protein complexes. It calculates the MMPBSA energy from molecular mechanics (MM), electrostatic contributions to solvation (PB) and the non-polar contributions to solvation (SA). Essentially, it combines the Poisson–Boltzmann equation and MD simulations to calculate solvation energy [52]. The g_mmpbsa tool in gromacs uses the single-trajectory MMPBSA method to post-process binding free energies from MD output files. It uses 100 frames at equal distances along the 20 ns trajectory files.

For non-covalent binding interactions in the aqueous phase the binding free energy, ΔG (bind,aq), can be calculated as: –.

2 $$ \Delta \mathrm{G}\ \left(\mathrm{bind},\mathrm{aq}\right)=\Delta \mathrm{G}\ \left(\mathrm{bind},\mathrm{vac}\right)+\Delta \mathrm{G}\ \left(\mathrm{bind},\mathrm{solv}\right) $$

where ΔG (bind,vac) is the binding free energy in vacuum, and ΔG (bind,solv) was the binding induced solvation free energy change: –.

3 $$ \Delta \mathrm{G}\ \left(\mathrm{bind},\mathrm{solv}\right)=\Delta \mathrm{G}\ \left(\mathrm{R}:\mathrm{L},\mathrm{solv}\right)-\Delta \mathrm{G}\ \left(\mathrm{R},\mathrm{solv}\right)-\Delta \mathrm{G}\ \left(\mathrm{L},\mathrm{solv}\right) $$

where ΔG (R:L,solv), ΔG (R,solv) and ΔG (L,solv) are solvation free energies for respectively, the bound complex, receptor and ligand.

## Supplementary Information

**Additional file 1: Table S1.** Binding energies and published SARS-Cov-2 data for 80 top ranked small molecule ligands. **Table S2.** Binding interactions with RdRP binding site for top 10 ranked drugs. **Table S3.** Top 20 natural drugs predicted to bind and inhibit RdRP. The drug descriptions are taken directly from DrugBank. **Figure S1.** LigPlot (left) and hydrophobic protein surface representation (right) of the main interactions between RdRP and ergotamine. **Figure S2.** LigPlot (left) and hydrophobic protein surface representation (right) of the main interactions between RdRP and bemcentinib.

## Abbreviations

ACE2: angiotensin converting enzyme 2
dsRNA: doubled stranded RNA
GPU: graphics processing unit
MD: molecular dynamics
MERS: Middle East respiratory syndrome
MMPBSA: Molecular Mechanics Poisson-Boltzmann Surface Area
M^pro^: SARS-CoV-2 main protease
PLpro: papain-like protease
PME: particle mesh Ewald
RdRP: RNA-dependent RNA polymerase
RMSD: root-mean-square deviation
SARS: severe acute respiratory syndrome

## References

[1] Dai W, Zhang B, Jiang XM, Su H, Li J, Zhao Y, et al. Structure-based design of antiviral drug candidates targeting the SARS-CoV-2 main protease. Science. 2020;368(6497):1331–5. 10.1126/science.abb4489. 32321856 10.1126/science.abb4489 PMC7179937

[2] Zhu W, Chen CZ, Gorshkov K, Xu M, Lo DC, Zheng W. RNA-dependent RNA polymerase as a target for COVID-19 drug discovery. SLAS Discov. 2020;25(10):1141–51. 10.1177/2472555220942123. 32660307 10.1177/2472555220942123 PMC7684788

[3] Guterres H, Im W. Improving protein-ligand docking results with high-throughput molecular dynamics simulations. J Chem Inf Model. 2020;60(4):2189–98. 10.1021/acs.jcim.0c00057. 32227880 10.1021/acs.jcim.0c00057 PMC7534544

[4] Winkler DA. Ligand entropy is hard but should not be ignored. J Chem Inf Model. 2020;60(10):4421–3. 10.1021/acs.jcim.0c01146. 33100015 10.1021/acs.jcim.0c01146

[5] Beg MA, Athar F. Anti-HIV and anti-HCV drugs are the putative inhibitors of RNA-dependent-RNA polymerase activity of nsp12 of the SARS-CoV- 2 (COVID-19). Pharm Pharmacol Int J. 2020;8(3):163–72. 10.15406/ppij.2020.08.00292.

[6] Cozac R, Medzhidov N, Yuki S. Predicting inhibitors for SARS-CoV-2 RNA-dependent RNA polymerase using machine learning and virtual screening}. arXiv. 2020:2006.06523.

[7] Dutta K, Shityakov S, Morozova O, Khalifa I, Zhang J, Zhu W, et al. Beclabuvir can inhibit the RNA-dependent RNA polymerase of newly emerged novel coronavirus (SARS-CoV-2). Preprints. 2020:2020030395. 10.20944/preprints202003.0395.v2.

[8] Shannon A, Le NT, Selisko B, Eydoux C, Alvarez K, Guillemot JC, et al. Remdesivir and SARS-CoV-2: structural requirements at both nsp12 RdRp and nsp14 exonuclease active-sites. Antivir Res. 2020;178:104793. 10.1016/j.antiviral.2020.104793. 32283108 10.1016/j.antiviral.2020.104793 PMC7151495

[9] Anastasiou IA, Eleftheriadou I, Tentolouris A, Tsilingiris D, Tentolouris N. In vitro data of current therapies for SARS-CoV-2. Curr Med Chem. 2020;27(27):4542–8. 10.2174/0929867327666200513075430. 32400323 10.2174/0929867327666200513075430

[10] Dyer O. Covid-19: Remdesivir has little or no impact on survival, WHO trial shows. Br Med J. 2020;371:m4057. 10.1136/bmj.m4057. 33077424 10.1136/bmj.m4057

[11] Elfiky AA. Ribavirin, remdesivir, sofosbuvir, galidesivir, and tenofovir against SARS-CoV-2 RNA dependent RNA polymerase (RdRp): a molecular docking study. Life Sci. 2020;253:117592. 10.1016/j.lfs.2020.117592. 32222463 10.1016/j.lfs.2020.117592 PMC7102646

[12] Ahmed S, Mahtarin R, Ahmed SS, Akter S, Islam MS, Mamun AA, et al. Investigating the binding affinity, interaction, and structure-activity-relationship of 76 prescription antiviral drugs targeting RdRp and Mpro of SARS-CoV-2. J Biomol Struct Dyn. 2020:1–16. 10.1080/07391102.2020.1796804. 10.1080/07391102.2020.1796804 PMC7441766 32720571

[13] Aouidate A, Ghaleb A, Chtita S, Aarjane M, Ousaa A, Maghat H, et al. Identification of a novel dual-target scaffold for 3CLpro and RdRp proteins of SARS-CoV-2 using 3D-similarity search, molecular docking, molecular dynamics and ADMET evaluation. J Biomol Struct Dyn. 2020;39(12):1–14. 10.1080/07391102.2020.1779130. 10.1080/07391102.2020.1779130 PMC7309310 32552534

[14] Banerjee S, Dey R, Seal S, Mondal KK, Bhattacharjee P. Identification of best suitable repurposed drugs considering mutational spectra at RdRp (nsp12), 3CLpro (nsp5) and PLpro (nsp3) of SARS-CoV-2 in indian population. Res Square. 2020. 10.21203/rs.3.rs-33879/v1.

[15] Heidary F, Gharebaghi R. Ivermectin: a systematic review from antiviral effects to COVID-19 complementary regimen. J Antibiot (Tokyo). 2020;73(9):593–602. 10.1038/s41429-020-0336-z. 32533071 10.1038/s41429-020-0336-z PMC7290143

[16] Caly L, Druce JD, Catton MG, Jans DA, Wagstaff KM. The FDA-approved drug ivermectin inhibits the replication of SARS-CoV-2 in vitro. Antivir Res. 2020;178:104787. 10.1016/j.antiviral.2020.104787. 32251768 10.1016/j.antiviral.2020.104787 PMC7129059

[17] Dixit A, Yadav R, Singh AV. Ivermectin: Potential role as repurposed drug for COVID-19. Malays J Med Sci. 2020;27(4):154–158. 10.21315/mjms2020.27.4.15 10.21315/mjms2020.27.4.15 PMC7444833 32863755

[18] Daghir Janabi AH. Effective anti-SARS-CoV-2 RNA dependent RNA polymerase drugs based on docking methods: The case of milbemycin, ivermectin, and baloxavir marboxil. Avicenna J Med Biotechnol. 2020;12(4):246–50. https://www.ncbi.nlm.nih.gov/pubmed/33014317. Published 2020/10/06. 33014317 PMC7502160

[19] Kalhor H, Sadeghi S, Abolhasani H, Kalhor R, Rahimi H. Repurposing of the approved small molecule drugs in order to inhibit SARS-CoV-2 s protein and human ACE2 interaction through virtual screening approaches. J Biomol Struct Dyn. 2020:1–16. 10.1080/07391102.2020.1824816. 10.1080/07391102.2020.1824816 PMC7576931 32969333

[20] Cho J, Lee YJ, Kim JH, Kim SI, Kim SS, Choi BS, et al. Antiviral activity of digoxin and ouabain against SARS-CoV-2 infection and its implication for COVID-19. npj Sci Rep. 2020;10(1):16200. 10.1038/s41598-020-72879-7. 10.1038/s41598-020-72879-7 PMC7530981 33004837

[21] Jeon S, Ko M, Lee J, Choi I, Byun SY, Park S, et al. Identification of antiviral drug candidates against SARS-CoV-2 from FDA-approved drugs. Antimicrob Agents Chemother. 2020;64(7):e00819–20. 10.1128/AAC.00819-20. 32366720 10.1128/AAC.00819-20 PMC7318052

[22] Bosch-Barrera J, Martin-Castillo B, Buxo M, Brunet J, Encinar JA, Menendez JA. Silibinin and SARS-CoV-2: dual targeting of host cytokine storm and virus replication machinery for clinical management of COVID-19 patients. J Clin Med. 2020;9(6):1770. 10.3390/jcm9061770. 32517353 10.3390/jcm9061770 PMC7356916

[23] Pokhrel R, Chapagain P, Siltberg-Liberles J. Potential RNA-dependent RNA polymerase inhibitors as prospective therapeutics against SARS-CoV-2. J Med Microbiol. 2020;69(6):864–73. 10.1099/jmm.0.001203. 32469301 10.1099/jmm.0.001203 PMC7451031

[24] Ahmad J, Ikram S, Ahmad F, Rehman IU, Mushtaq M. SARS-CoV-2 RNA dependent RNA polymerase (RdRp) - a drug repurposing study. Heliyon. 2020;6(7):e04502. 10.1016/j.heliyon.2020.e04502. 32754651 10.1016/j.heliyon.2020.e04502 PMC7377705

[25] Imami AS, O'Donovan SM, Creeden JF, Wu X, Eby H, McCullumsmith CB, et al. Oxytocin’s anti-inflammatory and proimmune functions in COVID-19: a transcriptomic signature-based approach. Physiol Genomics. 2020;52(9):401–7. 10.1152/physiolgenomics.00095.2020. 32809918 10.1152/physiolgenomics.00095.2020 PMC7877479

[26] Machitani M, Yasukawa M, Nakashima J, Furuichi Y, Masutomi K. RNA-dependent RNA polymerase, RdRP, a promising therapeutic target for cancer and potentially COVID-19. Cancer Sci. 2020;111(11):3976–84. 10.1111/cas.14618. 32805774 10.1111/cas.14618 PMC7461281

[27] Choudhury S, Moulick D, Saikia P, Mazumder MK. Evaluating the potential of different inhibitors on RNA-dependent RNA polymerase of severe acute respiratory syndrome coronavirus 2: A molecular modeling approach. Med J Arm Forc India. 2020: in press. 10.1016/j.mjafi.2020.05.005. 10.1016/j.mjafi.2020.05.005 PMC7261222 32836709

[28] Nitulescu GM, Paunescu H, Moschos SA, Petrakis D, Nitulescu G, Ion GND, et al. Comprehensive analysis of drugs to treat sarscov2 infection: mechanistic insights into current covid19 therapies (review). Int J Mol Med. 2020;46(2):467–88. 10.3892/ijmm.2020.4608. 32468014 10.3892/ijmm.2020.4608 PMC7307820

[29] Chandra A, Gurjar V, Qamar I, Singh N. Identification of potential inhibitors of SARS-COV-2 endoribonuclease (EndoU) from FDA approved drugs: a drug repurposing approach to find therapeutics for COVID-19, Journal of Biomolecular Structure and Dynamics. 2021;39(12):4201-11. 10.1080/07391102.2020.1775127. 10.1080/07391102.2020.1775127 PMC7298882 32462970

[30] Care UoIH. University of iowa virology research helps facilitate new clinical trial for COVID-19. Published 2020. https://www.eurekalert.org/pub_releases/2020-04/uoih-uoi042920.php. Accessed 7 Mar 2021

[31] Liu S, Lien CZ, Selvaraj P, Wang TT. Evaluation of 19 antiviral drugs against SARS-CoV-2 infection. bioRxiv. 2020:2020.04.29.067983. doi:10.1101/2020.04.29.067983.

[32] Dittmar M, Lee JS, Whig K, Segrist E, Li M, Jurado K, et al. Drug repurposing screens reveal FDA approved drugs active against SARS-Cov-2. bioRxiv. 2020:2020.06.19.161042. 10.1101/2020.06.19.161042.

[33] Xie X, Muruato AE, Zhang X, Lokugamage KG, Fontes-Garfias CR, Zou J, et al. A nanoluciferase SARS-CoV-2 for rapid neutralization testing and screening of anti-infective drugs for COVID-19. bioRxiv. 2020:2020.06.22.165712. 10.1101/2020.06.22.165712. 10.1038/s41467-020-19055-7 PMC7567097 33060595

[34] Yamamoto N, Matsuyama S, Hoshino T, Yamamoto N. Nelfinavir inhibits replication of severe acute respiratory syndrome coronavirus 2 in vitro. bioRxiv. 2020:2020.04.06.026476. 10.1101/2020.04.06.026476.

[35] Ko M, Jeon S, Ryu WS, Kim S. Comparative analysis of antiviral efficacy of FDA-approved drugs against SARS-CoV-2 in human lung cells. J Med Virol. 2021;93(3):1403–8. 10.1002/jmv.26397. 32767684 10.1002/jmv.26397 PMC7436731

[36] Milani M, Donalisio M, Bonotto RM, Schneider E, Arduino I, Boni F, et al. Combined in silico docking and in vitro antiviral testing for drug repurposing identified lurasidone and elbasvir as SARS-CoV-2 and hcov-oc43 inhibitors. bioRxiv. 2020:2020.11.12.379958. 10.1101/2020.11.12.379958.

[37] Vatansever EC, Yang K, Kratch KC, Drelich A, Cho C-C, Mellot DM, et al. Targeting the SARS-CoV-2 main protease to repurpose drugs for COVID-19. bioRxiv. 2020:2020.05.23.112235. 10.1101/2020.05.23.112235.

[38] Van Damme E, De Meyer S, Bojkova D, Ciesek S, Cinatl J, De Jonghe S, et al. In vitro activity of itraconazole against SARS-CoV-2. bioRxiv. 2020:2020.11.13.381194. 10.1101/2020.11.13.381194. 10.1002/jmv.26917 PMC8014624 33666253

[39] Nakajima K, Ogawa F, Sakai K, Uchiyama M, Oyama Y, Kato H, et al. A case of coronavirus disease 2019 treated with ciclesonide. Mayo Clin Proc. 2020;95(6):1296–7. 10.1016/j.mayocp.2020.04.007. 32402512 10.1016/j.mayocp.2020.04.007 PMC7158774

[40] Sales-Medina DF, Ferreira LRP, Romera LMD, Gonçalves KR, Guido RVC, Courtemanche G, et al. Discovery of clinically approved drugs capable of inhibiting SARS-CoV-2 in vitro infection using a phenotypic screening strategy and network-analysis to predict their potential to treat covid-19. bioRxiv. 2020:2020.07.09.196337. 10.1101/2020.07.09.196337.

[41] Rothlin RP, Vetulli HM, Duarte M, Pelorosso FG. Telmisartan as tentative angiotensin receptor blocker therapeutic for COVID-19. Drug Dev Res. 2020;81(7):768–70. 10.1002/ddr.21679. 32356926 10.1002/ddr.21679 PMC7267340

[42] Lo HS, Hui KPY, Lai H-M, Khan KS, Kaur S, Li Z, et al. Simeprevir suppresses SARS-CoV-2 replication and synergizes with remdesivir. bioRxiv. 2020:2020.05.26.116020. 10.1101/2020.05.26.116020.

[43] Drayman N, Jones KA, Azizi S-A, Froggatt HM, Tan K, Maltseva NI, et al. Drug repurposing screen identifies masitinib as a 3CLpro inhibitor that blocks replication of SARS-CoV-2 <em>in vitro</em>. bioRxiv. 2020:2020.08.31.274639. 10.1101/2020.08.31.274639.

[44] Weston S, Haupt R, Logue J, Matthews K, Frieman MB. FDA approved drugs with broad anti-coronaviral activity inhibit SARS-CoV-2 in vitro. bioRxiv. 2020:2020.03.25.008482. 10.1101/2020.03.25.008482.

[45] Xiao X, Wang C, Chang D, Wang Y, Dong X, Jiao T, et al. Identification of potent and safe antiviral therapeutic candidates against SARS-CoV-2. bioRxiv. 2020:2020.07.06.188953. 10.1101/2020.07.06.188953. 10.3389/fimmu.2020.586572 PMC7723961 33324406

[46] Pettersen EF, Goddard TD, Huang CC, Couch GS, Greenblatt DM, Meng EC, et al. UCSF chimera--a visualization system for exploratory research and analysis. J Comput Chem. 2004;25(13):1605–12. 10.1002/jcc.20084. 15264254 10.1002/jcc.20084

[47] Forli S, Huey R, Pique ME, Sanner MF, Goodsell DS, Olson AJ. Computational protein-ligand docking and virtual drug screening with the autodock suite. Nat Protoc. 2016;11(5):905–19. 10.1038/nprot.2016.051. 27077332 10.1038/nprot.2016.051 PMC4868550

[48] Laskowski RA, Swindells MB. Ligplot+: multiple ligand-protein interaction diagrams for drug discovery. J Chem Inf Model. 2011;51(10):2778–86. 10.1021/ci200227u. 21919503 10.1021/ci200227u

[49] Zoete V, Cuendet MA, Grosdidier A, Michielin O. Swissparam: a fast force field generation tool for small organic molecules. J Comput Chem. 2011;32(11):2359–68. 10.1002/jcc.21816. 21541964 10.1002/jcc.21816

[50] Abraham MJ, Murtola T, Schulz R, Páll S, Smith JC, Hessa B, et al. GROMACS; high performance molecular simulations through multi-level parallelism from laptops to supercomputers. SoftwareX. 2015;1–2:19–25. 10.1016/j.softx.2015.06.001.

[51] Paissoni C, Spiliotopoulos D, Musco G, Spitaleri A. GMXPBSA 2.1: a GROMACS tool to perform MM/PBSA and computational alanine scanning. Comp Phys Comm. 2015;186:105–7. 10.1016/j.cpc.2014.09.010.

[52] Baker NA, Sept D, Holst MJ, McCammon JA. The adaptive multilevel finite element solution of the poisson-boltzmann equation on massively parallel computers. IBM J Res Devel. 2001;45(3.4):427–38. 10.1147/rd.453.0427.

