# Supplementary material for: Computationally repurposed drugs and natural products against RNA dependent RNA polymerase as potential COVID-19 therapies
Source: Mol Biomed. 2021 Sep 20;2:28. doi: 10.1186/s43556-021-00050-3 (PMC8450033; doi:10.1186/s43556-021-00050-3)
Supplement: Supplementary file 1 — Additional file 1: Table S1. Binding energies and published SARS-Cov-2 data for 80 top ranked small molecule ligands. Table S2. Binding interactions with RdRP binding site for top 10 ranked drugs. Table S3. Top 20 natural drugs predicted to bind and inhibit RdRP. The drug descriptions are taken directly from DrugBank. Figure S1. LigPlot (left) and hydrophobic protein surface representation (right) of the main interactions between RdRP and ergotamine. Figure S2. LigPlot (left) and hydrophobic protein surface representation (right) of the main interactions between RdRP and bemcentinib. [file 43556_2021_50_MOESM1_ESM.docx]

**Computational screening of repurposed drugs and natural products against SARS-Cov-2 RdRP as potential COVID-19 therapies**

Sakshi Piplani^1-2^, Puneet Singh^1-2^, Nikolai Petrovsky^1-2^, David A. Winkler^3-6^

^1^ College of Medicine and Public Health, Flinders University, Bedford Park 5046, Australia

^2^ Vaxine Pty Ltd, 11 Walkley Avenue, Warradale 5046, Australia

^3^ La Trobe University, Kingsbury Drive, Bundoora 3042, Australia

^4^ Monash Institute of Pharmaceutical Sciences, Monash University, Parkville 3052, Australia

^5^ School of Pharmacy, University of Nottingham, Nottingham NG7 2RD. UK

^6^ CSIRO Data61, Pullenvale 4069, Australia

**Supplementary information**

| **Table S1**. Binding energies and published SARS-Cov-2 data for 80 top ranked small molecule ligands | | | | |
| --- | --- | --- | --- | --- |
|  | ChemBL (C)) or  Drugbank (D) ID | Name | ΔG_MMPBSA_  kcal/mol | SARS-CoV-2 data |
| 1 | C 3391662 | Paritaprevir | -54.3 | Predicted inhibitor of Mpro and RdRP.^1,2^ |
| 2 | C 1200633 | Ivermectin | -54.1 | IC_50_ of 2.2 - 2.8 µM in monkey kidney cells.^3-5^ |
| 3 | C 3126842 | Beclabuvir | -53.6 |  |
| 4 | C 3809489 | Bemcentinib | -46.2 | 10-40% protection at 50µM in Vero cells.^6^ IC_50_ of 100nM and CC_50_ of 4.7µM in human Huh7.5 cells and an IC_50_ of 470nM and CC_50_ was 1.6µM in Vero cells,^7^ investigational treatment for COVID-19 (www.clinicaltrialsregister.eu), predicted to bind to Mpro.^2^ |
| 5 | D 14761 | Remdesivir | -44.7 | In clinical trial for COVD-19, results equivocal,^8,9^ many in vitro reports e.g. IC_50_ of 1µM and CC_50_ of 275 µM in Vero cells,^10^ IC_50_ of 11.4 µM in Vero cells and IC_50_ of 1.3 µM in Calu-3 human lung cells,^11^ SARS-Cov-2 RdRP EC_50_ = 0.007 μM with IC_50_ of 1.7 µM (Vero), 0.3 µM (Calu-3) and 0.01 µM (human airway epithelial cells),^12^ computational prediction of RdRP inhibition,^13,14^ and Mpro inhibition. ^2^ |
| 6 | C 1751 | Digoxin | -41.2 | Predicted RdRP inhibitor,^15^ IC_50_ = 0.043 μM and CC_50_ >10µM in Vero cells,^16^ |
| 7 | D 09298 | Silibinin | -40.3 | Predicted RdRP inhibitor,^17,18^ |
| 8 | C 1236524 | Galidesvir | -40.3 | Clinical trials for COVID-19 and RdRP inhibitor,^19^predicted Mpro inhibitor,^20^ |
| 9 | C 1076263 | Setrobuvir | -40.0 | Predicted SARS-Cov-2 RdRP^13,21^ and Mpro inhibitor,^22^ |
| 10 | C 3707372 | Voxilaprevir | -39.5 | Experimental EC_50_ >10 µM and CC_50_ 16 µM in A549-hACE2 cells,^23^predicted RdRP^24^ and Mpro inhibitor.^25^ |
| 11 | C 2013174 | Vedroprevir | -38.5 | Predicted Mpro^26^and RdRP inhibitor,^27^ |
| 12 | C 1241348 | Faldaprevir | -38.1 | Predicted Mpro, ^28^ PLpro,^29^ and RdRP inhibitor,^27^ |
| 13 | C 413 | Sirolimus (Rapamycin) | -37.5 | Clinical trial for COVID-19,^30,31^ predicted Mpro, PLpro and spike inhibition,^32,33^ |
| 14 | C 3301668 | Carbetocin | -37.4 | Predicted RdRP ^34^and Mpro inhibitor,^35^ |
| 15 | D 01051 | Novobiocin | -37.3 | Predicted RdRP inhibition,^14^ |
| 16 | C 1683590 | Eribulin | -36.6 | Predicted RdRP^36^and 2′-O-ribose methyltransferase inhibitor.^37^ |
| 17 | C 442 | Ergotamine | -36.3 | Predicted IC_50_ of 190µM,^38^ predicted Mpro and RdRP inhibitor.^2,39^ |
| 18 | C 1957287 | Tegobuvir | -34.7 | SARS-Cov-2 EC_50_ = >10 and CC_50_ = 18 µM,^23^ predicted RdRP and Mpro inhibitor,^40-42^ |
| 19 | D 12466 | Favipiravir | -34.3 | Human trials for COVID-19,^9,43,44^ EC_50_ = 62 µM and CC_50_ =400 µM (Vero cells),^45^ predicted RdRP, helicase and Mpro inhibitor.^21,46,47^ |
| 20 | D 14850 | Deleobuvir | -34.2 | Predicted Mpro ^42^inhibitor. |
| 21 | C 297884 | Ciluprevir | -34.1 | Predicted Mpro ^48^ inhibitor, SARS-Cov-2 Mpro IC_50_ = 21 µM,^49^ |
| 22 | C 1200649 | Quinupristin | -33.8 | Predicted RdRP inhibitor ^33^ |
| 23 | D 01764 | Dalfopristin | -33.7 | Predicted RdRP inhibitor ^33^ |
| 24 | D 04703 | Hesperidin | -33.4 | Predicted Mpro ^50-52^ and RdRP inhibitor ^50^ |
| 25 | C 3545363 | Glecaprevir | -32.9 | Predicted Mpro ^53^ and RdRP inhibitor ^24^ |
| 26 | D 00224 | Indinavir | -32.6 | Predicted Mpro and RdRP inhibitor, ^24^ SARS-Cov-2 EC_50_ >10 µM and CC_50_ >50 µM (A549-hACE2 cells) ^23^ In vitro EC_50_: 59 μM; CC_50_ >81 μM (Vero cells),^54^ |
| 27 | D 06290 | Simeprevir | -31.7 | In vitro SARS-Cov-2 EC_50_ = 4 µM and CC_50_ 19 µM (Vero cells), in Vitro Mpro inhibition IC_50_ = 10 µM, negligible PLpro and RdRP enzyme inhibition,^55^ predicted Mpro and RdRP inhibitor.^2,56^ |
| 28 | C 461101 | Eltrombopag | -31.6 | Predicted Mpro ^2,39^ and RdRP inhibitor,^39,40^ in vitro SARS-Cov-2 IC_50_ = 8 µM and CC_50_ >50 µM (Vero cells),^57^ IC_50_ = 8 µM (Vero and Calu-3 cells) ^11^ |
| 29 | D 01126 | Dutasteride | -31.6 | Predicted Mpro and RdRP inhibitor and regulates expression of transmembrane serine protease 2 (TMPRSS2),^39^ |
| 30 | C 3039514 | Elbasvir | -31.6 | SARS-Cov-2 in vitro EC_50_ = 23 µM (Huh7-hACE2 cells),^58^predicted Mpro ^2^ and RdRP inhibition.^59^ |
| 31 | C 44657 | Etoposide | -30.4 | Predicted Mpro ^60,61^ |
| 32 | D 06638 | Quarfloxin | -30.2 | Predicted Mpro, human ACE2,^2,62^ and PLpro inhibitor.^63^ |
| 33 | D 00445 | Epirubicin | -29.9 | Predicted Mpro,^39^ |
| 34 | C 3833385 | Ruzasvir | -28.9 | Predicted Mpro, RdRP inhibitor^1,2^ |
| 35 | D 11753 | Rifamycin | -28.5 | Predicted Mpro and RdRP inhibitor.^2,64^ |
| 36 | C 1017 | Telmisartan | -28.0 | Predicted human ACE2 blocker and clinical trial for COVID-19 treatment (NCT04356495).^65^predicted Mpro and RdRP inhibitor.^66,67^ |
| 37 | D 03523 | Brequinar | -28.0 | Experimental SARS-Cov-2 EC_50_ = 0.3 µM, CC_50_ > 50 µM (Vero E6 cells),^68^ |
| 38 | D 00619 | Imatinib | -27.7 | Experimental 80% reduction in Mpro activity at 10 µM, EC_50_ = 8 µM (A549 cells),^69^predicted Mpro inhibitor,^70^ experimental SARS-Cov-2 IC_50_ = 3-5µM, CC_50_ >30 µM,^71^ |
| 39 | D 00872 | Conivaptan | -27.6 | Predicted Mpro and RdRP inhibitor,^39,72^ experimental SARS-COV-2 IC_50_ = 10 µM (Vero cells),^73^EC_50_ = 4µM (ACE2-A549 cells), SARS-Cov-2 Mpro IC_50_ ~ 10µM (293T cells),^69^ |
| 40 | D 08901 | Ponatinib | -27.1 | Experimental SARS-Cov-2 EC_50_ = 1 µM, CC_50_ 9 µM (HEK-293T cells),^58^ predicted SARS-Cov-2 spike inhibitor.^74^ |
| 41 | C 1660 | Rifapentine | -27.1 | Predicted RdRP^64,75^ and Mpro inhibitor.^76^ |
| 42 | D 06144 | Sertindole | -26.6 | Predicted Mpro inhibition, but negligible experimental SARS-Cov-2 Mpro inhibition.^77^ |
| 43 | C 2063090 | Grazoprevir | -26.5 | Experimental SARS-CoV-2 inhibition with EC_50_ = 16 μM (CC_50_ value >100 μM, Vero E6 cells),^58^ predicted Mpro^78^ and RdRP inhibitor.^24,40^ |
| 44 | D 01167 | Itraconazole | -26.4 | Predicted Mpro^51^ and RdRP inhibition,^15^ experimental SARS-Cov-2 Mpro EC_50_ = 110 µM.^77^and SARS-Cov-2 EC_50_ = 2.3 μM (human Caco-2 cells).^79^ |
| 45 | C 2103975 | Vapreotide | -26.2 | Predicted Mpro^80^ inhibition |
| 46 | D 06733 | Bafilomycin A1 | -25.9 | Experimental SARS-Cov-2 EC_50_ > 50µM and CC_50_ > 50 µM,^68^ |
| 47 | C 1738757 | Rebastinib | -25.7 | Predicted Mpro^2^ and 2’-O-ribose methyltransferase^81,82^ inhibitor. |
| 48 | D 06448 | Lonafarnib | -25.3 | Predicted RdRP^40^ PLpro and Mpro^25,63^ inhibitor. |
| 49 | C 493 | Bromocriptine | -25.3 | Predicted Mrpo^2^ and NSP14 inhibitor.^18^ |
| 50 | D 01177 | Idarubicin | -25.2 | Predicted RdRP and Mpro inhibitor.^66,83^ Weak in vitro Mpro activity IC_50_ = 250−600µM. ^84^ |
| 51 | C 608533 | Midostaurin | -25.0 | Predicted Mpro,^2^ and spike inhibitor.^85^ |
| 52 | D 01698 | Rutin | -25.0 | Predicted RdRP and Mpro inhibitor.^86^ |
| 53 | C 2103882 | Tivantinib | -24.8 | Predicted Mpro and 2 2’-O-methyltransferase inhibitor.^42,82^ |
| 54 | C 490672 | Filibuvir | -24.6 | Predicted RdRP^40^and Mpro inhibitor.^42^ |
| 55 | C 1983268 | Entrectinib | -23.8 | Predicted SARS-Cov-2 2’-O-methyltransferase inhibitor.^81^ |
| 56 | C 1429 | Desmopressin | -23.7 | Predicted SARS-Cov-2 spike,^87^helicase,^61^spike,^88^and 2’- O-methyltransferase inhibitor.^89^ |
| 57 | C 2106409 | Elsamitrucin | -22.6 | Predicted Mpro inhibitor.^90^ |
| 58 | D 11618 | Zorubicin | -22.3 | Predicted inhibitor of SARS-Cov-2 spike. ^91^ |
| 59 | C 3039525 | Golvatinib | -22.2 | Predicted SARS-Cov-2 RDRP NSP12‐NSP7 interface,^40^ NSP14 SAM-dependent N7-methyl transferase, ^18^ Mpro,^2^ and 2'-O-Ribose Methyltransferase Nsp16^82^ |
| 60 | C 532 | Erythromycin | -21.6 | Predicted SARS-Cov-2 Mpro inhibitor.^92^ |
| 61 | D 01092 | Ouabain | -21.3 | Predicted SARS-Cov-2 RdRP and Mpro inhibitor.^15,42^ |
| 62 | C 3318007 | Pimodivir | -21.2 | Predicted SARS-Cov-2 Mpro inhibitor. ^42,93^ |
| 63 | D 04785 | Streptolydigin | -20.8 | Predicted SARS-Cov-2 RdRP inhibition.^64^ |
| 64 | D 11616 | Pirarubicin | -20.3 | … |
| 65 | C 2104415 | Moxidectin | -20.2 | In vitro IC_50_ = 3µM in LLC-MK2 cells, ^73^ predicted inhibitor of Mpro, RdRP and human ACE2 receptor.^2,94^ |
| 66 | C 3137309 | Venetoclax | -20.1 | Inactive in SARS‐CoV‐2 CPE assay,^95^ predicted Mpro inhibitor^60^ |
| 67 | D 00549 | Zafirlukast | -20.0 | Predicted inhibitor of SARS-Cov-2 Mpro, spike, and 2'-O-methyltransferases.^2,37,91^ |
| 68 | C 4093031 | BMS-929075 | -19.8 | … |
| 69 | D 01267 | Paliperidone | -19.4 | Predicted inhibitor of SARS-Cov-2 RdRP and 2'-O-methyltransferases.^39,82^ |
| 70 | C 1951095 | Eravacycline | -18.8 | Predicted inhibitor of SARS-CoV-2 Mpro ^2,96^ RdRP (NSP12), and human ACE2 receptor.^94^ |
| 71 | C 372795 | Streptomycin | -18.4 | … |
| 72 | D 09280 | Lumacaftor | -17.8 | Predicted to be a SARS-Cov-2 spike protein, ^85^ Mpro^97^ and helicase inhibitor.^98^ |
| 73 | C 3989904 | Cethromycin | -17.0 | … |
| 74 | D 01254 | Dasatinib | -16.9 | Active against SARS and MERS at low µM in vitro.^99^ Predicted to bind to Mpro. ^100^ |
| 75 | D 01410 | Ciclesonide | -16.7 | In vitro EC_90_ for SARS-CoV-2 of 5µM in Vero cells and 0.55µM in differentiated human bronchial tracheal epithelial cells, blocks viral RNA replication, supresses replication of 15 mutants by >90% ^57,101^Used to treat COVID-19 patients. ^102^ |
| 76 | C 4297453 | Zalypsis | -16.0 | … |
| 77 | C 444172 | Zosuquidar | -15.7 | Predicted to target 2'-O-ribose methyltransferase Nsp16 of SARS-CoV-2 |
| 78 | C 1471 | Aprepitant | -15.6 | Predicted to bind to SARS-Cov-2 Mpro ^80^ |
| 79 | D 03325 | Tyrosyladenylate | -13.6 | Predicted to bind to SARS-CoV-2 Nsp16 2’-O-MTase ^103^ |
| 80 | C 408 | Troglitazone | -13.2 | Predicted to bind to SARS-Cov-2 spike^91^ |

**References**

1. Cozac R, Medzhidov N, Yuki S. Predicting inhibitors for SARS-CoV-2 RNA-dependent RNA polymerase using machine learning and virtual screening}. *arXiv.* 2020:2006.06523.

2. Piplani S, Singh P, Petrovsky N, Winkler DA. Computational screening of repurposed drugs and natural products against SARS-Cov-2 main protease as potential COVID-19 therapies. *Antiviral Res.* 2021:submitted.

3. Heidary F, Gharebaghi R. Ivermectin: A systematic review from antiviral effects to COVID-19 complementary regimen. *J Antibiot (Tokyo).* 2020;73(9):593-602. doi:10.1038/s41429-020-0336-z

4. Simsek Yavuz S, Unal S. Antiviral treatment of COVID-19. *Turk J Med Sci.* 2020;50(SI-1):611-619. doi:10.3906/sag-2004-145

5. Anastasiou IA, Eleftheriadou I, Tentolouris A, Tsilingiris D, Tentolouris N. In vitro data of current therapies for SARS-CoV-2. *Curr Med Chem.* 2020;27(27):4542-4548. doi:10.2174/0929867327666200513075430

6. Liu S, Lien CZ, Selvaraj P, Wang TT. Evaluation of 19 antiviral drugs against SARS-CoV-2 infection. *bioRxiv.* 2020:2020.04.29.067983. doi:10.1101/2020.04.29.067983.

7. Dittmar M, Lee JS, Whig K, Segrist E, Li M, Jurado K, Samby K, et al. Drug repurposing screens reveal FDA approved drugs active against SARS-CoV-2. *bioRxiv.* 2020:2020.06.19.161042. doi:10.1101/2020.06.19.161042.

8. Dyer O. COVID-19: Remdesivir has little or no impact on survival, WHO trial shows. *Br Med J.* 2020;371:m4057. doi:10.1136/bmj.m4057

9. Sreekanth Reddy O, Lai WF. Tackling COVID-19 using remdesivir and favipiravir as therapeutic options. *ChemBioChem.* 2021;22(6):939-948. doi:10.1002/cbic.202000595

10. Pizzorno A, Padey B, Dubois J, Julien T, Traversier A, Duliere V, et al. In vitro evaluation of antiviral activity of single and combined repurposable drugs against SARS-CoV-2. *Antiviral Res.* 2020;181:104878. doi:10.1016/j.antiviral.2020.104878

11. Ko M, Jeon S, Ryu WS, Kim S. Comparative analysis of antiviral efficacy of FDA-approved drugs against SARS-CoV-2 in human lung cells. *J Med Virol.* 2021;93(3):1403-1408. doi:10.1002/jmv.26397

12. Pruijssers AJ, George AS, Schafer A, Leist SR, Gralinksi LE, Dinnon KH 3rd, et al. Remdesivir inhibits SARS-CoV-2 in human lung cells and chimeric SARS-CoV expressing the SARS-CoV-2 RNA polymerase in mice. *Cell Rep.* 2020;32(3):107940. doi:10.1016/j.celrep.2020.107940

13. Elfiky AA. Ribavirin, remdesivir, sofosbuvir, galidesivir, and tenofovir against SARS-CoV-2 RNA dependent RNA polymerase (RdRp): A molecular docking study. *Life Sci.* 2020;253:117592. doi:10.1016/j.lfs.2020.117592

14. Choudhury S, Moulick D, Saikia P, Mazumder MK. Evaluating the potential of different inhibitors on RNA-dependent RNA polymerase of severe acute respiratory syndrome coronavirus 2: A molecular modeling approach. *Med J Arm Forc India.* 2020:in press. doi:10.1016/j.mjafi.2020.05.005

15. Dey SK, Saini M, Dhembla C, Bhatt S, Rajesh AS, Anand V, Das HK, Kundu S. Suramin, penciclovir and anidulafungin bind nsp12, which governs the RNA-dependent-RNA polymerase activity of SARS-CoV-2, with higher interaction energy than remdesivir, indicating potential in the treatment of COVID-19 infection. *OSF Preprints.* 2020:urxwh. doi:10.31219/osf.io/urxwh.

16. Cho J, Lee YJ, Kim JH, Kim SI, Kim SS, Choi BS, et al. Antiviral activity of digoxin and ouabain against SARS-CoV-2 infection and its implication for COVID-19. *npj Sci Rep.* 2020;10(1):16200. doi:10.1038/s41598-020-72879-7

17. Bosch-Barrera J, Martin-Castillo B, Buxo M, Brunet J, Encinar JA, Menendez JA. Silibinin and SARS-CoV-2: Dual targeting of host cytokine storm and virus replication machinery for clinical management of COVID-19 patients. *J Clin Med.* 2020;9(6):1770. doi:10.3390/jcm9061770

18. Liu C, Zhu X, Lu Y, Zhang X, Jia X, Yang T. Potential treatment of chinese and western medicine targeting nsp14 of SARS-CoV-2. *J Pharm Anal.* 2020:in press. doi:10.1016/j.jpha.2020.08.002

19. Gil C, Ginex T, Maestro I, Nozal V, Barrado-Gil L, Cuesta-Geijo MA, et al. COVID-19: Drug targets and potential treatments. *J Med Chem.* 2020;63(21):12359-12386. doi:10.1021/acs.jmedchem.0c00606

20. Kumar S, Sharma PP, Shankar U, Kumar D, Joshi SK, Pena L, et al. Discovery of new hydroxyethylamine analogs against 3cl(pro) protein target of SARS-CoV-2: Molecular docking, molecular dynamics simulation, and structure-activity relationship studies. *J Chem Inf Model.* 2020;60(12):5754-5770. doi:10.1021/acs.jcim.0c00326

21. Elfiky AA. SARS-CoV-2 RNA dependent RNA polymerase (RdRp) targeting: An in silico perspective. *J Biomol Struct Dyn.* 2020:1-9. doi:10.1080/07391102.2020.1761882

22. Mosquera-Yuqui F, Lopez-Guerra N, Moncayo-Palacio EA. Targeting the 3CLpro and RdRp of SARS-CoV-2 with phytochemicals from medicinal plants of the Andean region: Molecular docking and molecular dynamics simulations. *J Biomol Struct Dyn.* 2020:1-14. doi:10.1080/07391102.2020.1835716

23. Xie X, Muruato AE, Zhang X, Lokugamage KG, Fontes-Garfias CR, Zou J, et al. A nanoluciferase SARS-CoV-2 for rapid neutralization testing and screening of anti-infective drugs for COVID-19. *bioRxiv.* 2020:2020.06.22.165712. doi:10.1101/2020.06.22.165712.

24. Indu P, Rameshkumar MR, Arunagirinathan N, Al-Dhabi NA, Valan Arasu M, Ignacimuthu S. Raltegravir, indinavir, tipranavir, dolutegravir, and etravirine against main protease and RNA-dependent RNA polymerase of SARS-CoV-2: A molecular docking and drug repurposing approach. *J Infect Pub Health.* 2020;13(12):1856-1861. doi:10.1016/j.jiph.2020.10.015

25. Ray AK, Gupta PSS, Panda SK, Biswal S, Rana MK. Repurposing of FDA approved drugs for the identification of potential inhibitors of SARS-CoV-2 main protease. *ChemRxiv.* 2020:chemrxiv.12278066.v1. doi:10.26434/chemrxiv.12278066.v1.

26. Shah B, Modi P, Sagar SR. In silico studies on therapeutic agents for COVID-19: Drug repurposing approach. *Life Sci.* 2020;252:117652. doi:10.1016/j.lfs.2020.117652

27. Cozac R, Medzhidov N, Yuk S. Predicting inhibitors for SARS-CoV-2 RNA-dependent RNA polymerase using machine learning and virtual screening. *arXiv.* 2020:2006.06523.

28. Eleftheriou P, Amanatidou D, Petrou A, Geronikaki A. In silico evaluation of the effectivity of approved protease inhibitors against the main protease of the novel SARS-CoV-2 virus. *Molecules.* 2020;25(11):2529. doi:10.3390/molecules25112529

29. Bagherzadeh K, Azizian H, Daneshvarnejad K, Abbasinazari M. In silico repositioning for dual inhibitor discovery of SARS-CoV-2 (COVID- 19) 3c-like protease and papain-like peptidase. *Preprints.* 2020:202004.0084.v1. doi:10.20944/preprints202004.0084.v1.

30. Nitulescu GM, Paunescu H, Moschos SA, Petrakis D, Nitulescu G, Ion GND, et al. Comprehensive analysis of drugs to treat SARS-CoV-2 infection: Mechanistic insights into current covid19 therapies (review). *Int J Mol Med.* 2020;46(2):467-488. doi:10.3892/ijmm.2020.4608

31. Wu R, Wang L, Kuo HD, Shannar A, Peter R, Chou PJ, et al. An update on current therapeutic drugs treating COVID-19. *Curr Pharmacol Rep.* 2020:1-15. doi:10.1007/s40495-020-00216-7

32. Mall R, Elbasir A, Al Meer H, Chawla S, Ullah E. Data-driven drug repurposing for COVID-19. *ChemRxiv.*chemrxiv.12661103.v1. doi:10.26434/chemrxiv.12661103.v1.

33. Pokhrel R, Chapagain P, Siltberg-Liberles J. Potential RNA-dependent RNA polymerase inhibitors as prospective therapeutics against SARS-CoV-2. *J Med Microbiol.* 2020;69(6):864-873. doi:10.1099/jmm.0.001203

34. Ahmad J, Ikram S, Ahmad F, Rehman IU, Mushtaq M. SARS-CoV-2 RNA dependent RNA polymerase (RdRp) - a drug repurposing study. *Heliyon.* 2020;6(7):e04502. doi:10.1016/j.heliyon.2020.e04502

35. Farag A, Wang P, Boys IN, Eitson JL, Ohlson MB, Fan W, et al. Identification of atovaquone, ouabain and mebendazole as FDA approved drugs targeting SARS-CoV-2. *ChemRxiv.* 2020:/chemrxiv.12003930.v4. doi:10.26434/chemrxiv.12003930.v4.

36. Hosseini M, Chen W, Wang C. Computational molecular docking and virtual screening revealed promising SARS-CoV-2 drugs. . *ChemRxiv.* 2020:chemrxiv.12237995.v1. doi:https://doi.org/10.26434/chemrxiv.12237995.v1.

37. Sharma K, Morla S, Goyal A, Kumar S. Computational guided drug repurposing for targeting 2'-o-ribose methyltransferase of SARS-CoV-2. *Life Sci.* 2020;259:118169. doi:10.1016/j.lfs.2020.118169

38. Chandra A, Gurjar V, Qamar I, Singh N. Identification of potential inhibitors of SARS-CoV-2 endoribonuclease (EndoU) from FDA approved drugs: A drug repurposing approach to find therapeutics for COVID-19. *J Biomol Struct Dyn.* 2020 (just-accepted):1-11. doi:10.1080/07391102.2020.1775127

39. Gul S, Ozcan O, Asar S, Okyar A, Baris I, Kavakli IH. In silico identification of widely used and well-tolerated drugs as potential SARS-CoV-2 3C-like protease and viral RNA-dependent RNA polymerase inhibitors for direct use in clinical trials. *J Biomol Struct Dyn.* 2020:1-20. doi:10.1080/07391102.2020.1802346

40. Ruan Z, Liu C, Guo Y, He Z, Huang X, Jia X, et al. SARS-CoV-2 and SARS-CoV: Virtual screening of potential inhibitors targeting RNA-dependent RNA polymerase activity (nsp12). *J Med Virol.* 2021;93(1):389-400. doi:10.1002/jmv.26222

41. Dutta K, Shityakov S, Morozova O, Khalifa I, Zhang J, Zhu W, et al. Beclabuvir can inhibit the RNA-dependent RNA polymerase of newly emerged novel coronavirus (SARS-CoV-2). *Preprints.* 2020:2020030395. doi:10.20944/preprints202003.0395.v2.

42. Peterson L. In silico molecular dynamics docking of drugs to the inhibitory active site of SARS-CoV-2 protease and their predicted toxicology and ADME. *ChemRxiv.* 2020:chemrxiv.12155523.v1. doi:10.26434/chemrxiv.12155523.v1.

43. Costanzo M, De Giglio MAR, Roviello GN. SARS-CoV-2: Recent reports on antiviral therapies based on lopinavir/ritonavir, darunavir/umifenovir, hydroxychloroquine, remdesivir, favipiravir and other drugs for the treatment of the new coronavirus. *Curr Med Chem.* 2020;27(27):4536-4541. doi:10.2174/0929867327666200416131117

44. Cai Q, Yang M, Liu D, Chen J, Shu D, Xia J, et al. Experimental treatment with favipiravir for COVID-19: An open-label control study. *Eng.* 2020;6(10):1192-1198. doi:10.1016/j.eng.2020.03.007

45. Wang M, Cao R, Zhang L, Yang X, Liu J, Xu M, et al Remdesivir and chloroquine effectively inhibit the recently emerged novel coronavirus (2019-nCov) in vitro. *Cell Res.* 2020;30(3):269-271. doi:10.1038/s41422-020-0282-0

46. Borgio JF, Alsuwat HS, Al Otaibi WM, Ibrahim AM, Almandil NB, Al Asoom LI, et al. State-of-the-art tools unveil potent drug targets amongst clinically approved drugs to inhibit helicase in SARS-CoV-2. *Arch Med Sci.* 2020;16(3):508-518. doi:10.5114/aoms.2020.94567

47. Sencanski M, Perovic V, Pajovic SB, Adzic M, Paessler S, Glisic S. Drug repurposing for candidate SARS-CoV-2 main protease inhibitors by a novel in silico method. *Molecules.* 2020;25(17):3830. doi:10.3390/molecules25173830

48. Chakraborti S, Bheemireddy S, Srinivasan N. Repurposing drugs against the main protease of SARS-CoV-2: Mechanism-based insights supported by available laboratory and clinical data. *Molecular omics.* 2020;16:474-491. doi:10.1039/d0mo00057d

49. Baker JD, Uhrich RL, Kraemer GC, Love JE, Kraemer BC. A drug repurposing screen identifies hepatitis Cantivirals as inhibitors of the SARS-CoV-2 main protease. *bioRxiv.* 2020:2020.07.10.197889. doi:10.1101/2020.07.10.197889.

50. Singh S, Sk MF, Sonawane A, Kar P, Sadhukhan S. Plant-derived natural polyphenols as potential antiviral drugs against SARS-CoV-2 via RNA-dependent RNA polymerase (RdRp) inhibition: An in-silico analysis. *J Biomol Struct Dyn.* 2020:1-16. doi:10.1080/07391102.2020.1796810

51. Das S, Sarmah S, Lyndem S, Singha Roy A. An investigation into the identification of potential inhibitors of SARS-CoV-2 main protease using molecular docking study. *J Biomol Struct Dyn.* 2020:1-11. doi:10.1080/07391102.2020.1763201

52. Joshi RS, Jagdale SS, Bansode SB, Shankar SS, Tellis MB, Pandya VK, et al. Discovery of potential multi-target-directed ligands by targeting host-specific SARS-CoV-2 structurally conserved main protease. *J Biomol Struct Dyn.* 2020:1-16. doi:10.1080/07391102.2020.1760137

53. Fischer A, Sellner M, Neranjan S, Smiesko M, Lill MA. Potential inhibitors for novel coronavirus protease identified by virtual screening of 606 million compounds. *Int J Mol Sci.* 2020;21(10):3626. doi:10.3390/ijms21103626

54. Yamamoto N, Matsuyama S, Hoshino T, Yamamoto N. Nelfinavir inhibits replication of severe acute respiratory syndrome coronavirus 2 in vitro. *bioRxiv.* 2020:2020.04.06.026476. doi:10.1101/2020.04.06.026476.

55. Lo HS, Hui KPY, Lai H-M, Khan KS, Kaur S, Li Z, et al. Simeprevir suppresses SARS-CoV-2 replication and synergizes with remdesivir. *bioRxiv.* 2020:2020.05.26.116020. doi:10.1101/2020.05.26.116020.

56. Ahmed S, Mahtarin R, Ahmed SS, Akter S, Islam MS, Mamun AA, et al. Investigating the binding affinity, interaction, and structure-activity-relationship of 76 prescription antiviral drugs targeting RdRp and Mpro of SARS-CoV-2. *J Biomol Struct Dyn.* 2020:1-16. doi:10.1080/07391102.2020.1796804

57. Jeon S, Ko M, Lee J, Choi I, Byun SY, Park S, et al. Identification of antiviral drug candidates against SARS-CoV-2 from FDA-approved drugs. *Antimicrob Agents Chemother.* 2020;64(7):e00819-00820. doi:10.1128/AAC.00819-20

58. Milani M, Donalisio M, Bonotto RM, Schneider E, Arduino I, Boni F, et al. Combined in silico docking and in vitro antiviral testing for drug repurposing identified lurasidone and elbasvir as SARS-CoV-2 and hCov-OC43 inhibitors. *bioRxiv.* 2020:2020.11.12.379958. doi:10.1101/2020.11.12.379958.

59. Beg MA, Athar F. Anti-HIV and anti-HCV drugs are the putative inhibitors of RNA-dependent-RNA polymerase activity of nsp12 of the SARS-CoV- 2 (COVID-19). . *Pharm Pharmacol Int J.* 2020;8(3):163‒172. doi: 10.15406/ppij.2020.08.00292

60. Chen YW, Yiu C-PB, Wong K-Y. Prediction of the SARS-CoV-2 (2019-nCov) 3C-like protease (3CL pro) structure: Virtual screening reveals velpatasvir, ledipasvir, and other drug repurposing candidates. *F1000Research.* 2020;9:f1000research.22457.2. doi:10.12688/f1000research.22457.2.

61. Anwar MU, Adnan F, Abro A, Khan MR, Rehman AU, Osama M, et al. Combined deep learning and molecular docking simulations approach identifies potentially effective FDA approved drugs for repurposing against SARS-CoV-2. *ChemRxiv.* 2020:chemrxiv.12227363.v1. doi:10.26434/chemrxiv.12227363.v1.

62. Alexpandi R, De Mesquita JF, Pandian SK, Ravi AV. Quinolines-based SARS-CoV-2 3CLpro and RdRp inhibitors and spike-RDB-ACE2 inhibitor for drug-repurposing against COVID-19: An in silico analysis. *Front Microbiol.* 2020;11:1796. doi:10.3389/fmicb.2020.01796

63. Murugan NA, Kumar S, Jeyakanthan J, Srivastava V. Searching for target-specific and multi-targeting organics for covid-19 in the drugbank database with a double scoring approach. *npj Sci Rep.* 2020;10(1):19125. doi:10.1038/s41598-020-75762-7

64. Elkarhat Z, Charoute H, Elkhattabi L, Barakat A, Rouba H. Potential inhibitors of SARS-CoV-2 RNA dependent RNA polymerase protein: Molecular docking, molecular dynamics simulations and mm-pbsa analyses. *J Biomol Struct Dyn.* 2020:1-14. doi:10.1080/07391102.2020.1813628

65. Rothlin RP, Vetulli HM, Duarte M, Pelorosso FG. Telmisartan as tentative angiotensin receptor blocker therapeutic for COVID-19. *Drug Dev Res.* 2020;81(7):768-770. doi:10.1002/ddr.21679

66. Jimenez-Alberto A, Ribas-Aparicio RM, Aparicio-Ozores G, Castelan-Vega JA. Virtual screening of approved drugs as potential SARS-CoV-2 main protease inhibitors. *Comput Biol Chem.* 2020;88:107325. doi:10.1016/j.compbiolchem.2020.107325

67. Ivanov J, Polshakov D, Kato-Weinstein J, Zhou Q, Li Y, Granet R, et al. Quantitative structure-activity relationship machine learning models and their applications for identifying viral 3CLpro- and RdRp-targeting compounds as potential therapeutics for COVID-19 and related viral infections. *ACS Omega.* 2020;5(42):27344-27358. doi:10.1021/acsomega.0c03682

68. Sales-Medina DF, Ferreira LRP, Romera LMD, Gonçalves KR, Guido RVC, Courtemanche G, et al. Discovery of clinically approved drugs capable of inhibiting SARS-CoV-2 in vitro infection using a phenotypic screening strategy and network-analysis to predict their potential to treat covid-19. *bioRxiv.* 2020:2020.07.09.196337. doi:10.1101/2020.07.09.196337.

69. Drayman N, Jones KA, Azizi S-A, Froggatt HM, Tan K, Maltseva NI, et al. Drug repurposing screen identifies masitinib as a 3CLpro inhibitor that blocks replication of SARS-CoV-2 in vitro. *bioRxiv.* 2020:2020.08.31.274639. doi:10.1101/2020.08.31.274639.

70. Kumar D, Chandel V, Raj S, Rathi B. In silico identification of potent FDA approved drugs against coronavirus COVID-19 main protease: A drug repurposing approach. *2020.* 2020;7(3):10. http://pubs.iscience.in/journal/index.php/cbl/article/view/1033. Published 2020-03-25.

71. Weston S, Haupt R, Logue J, Matthews K, Frieman MB. FDA approved drugs with broad anti-coronaviral activity inhibit SARS-CoV-2 in vitro. *bioRxiv.* 2020:2020.03.25.008482. doi:10.1101/2020.03.25.008482.

72. Khater S, Dasgupta N, Das G. Combining SARS-cov-2 proofreading exonuclease and RNA-dependent RNA polymerase inhibitors as a strategy to combat COVID-19: A high-throughput in silico screen. *OSF Preprints.* 2020:osf.io/7x5ek. doi:10.31219/osf.io/7x5ek.

73. Xiao X, Wang C, Chang D, Wang Y, Dong X, Jiao T, et al. Identification of potent and safe antiviral therapeutic candidates against SARS-CoV-2. *bioRxiv.* 2020:2020.07.06.188953. doi:10.1101/2020.07.06.188953.

74. Mulgaonkar N, Wang H, Mallawarachchi S, Fernando S, Martina B, Ruzek D. BCR-ABL tyrosine kinase inhibitor imatinib as a potential drug for COVID-19. *bioRxiv.* 2020:2020.06.18.158196. doi:10.1101/2020.06.18.158196.

75. Parvez MSA, Karim MA, Hasan M, Jaman J, Karim Z, Tahsin T, et al. Prediction of potential inhibitors for RNA-dependent RNA polymerase of SARS-CoV-2 using comprehensive drug repurposing and molecular docking approach. *Int J Biol Macromol.* 2020;163:1787-1797. doi:10.1016/j.ijbiomac.2020.09.098

76. Joshi T, Joshi T, Pundir H, Sharma P, Mathpal S, Chandra S. Predictive modeling by deep learning, virtual screening and molecular dynamics study of natural compounds against SARS-CoV-2 main protease. *J Biomol Struct Dyn.* 2020:1-19. doi:10.1080/07391102.2020.1802341

77. Vatansever EC, Yang K, Kratch KC, Drelich A, Cho C-C, Mellot DM,et al. Targeting the SARS-CoV-2 main protease to repurpose drugs for COVID-19. *bioRxiv.* 2020:2020.05.23.112235. doi:10.1101/2020.05.23.112235.

78. Hakmi M, Bouricha E, Kandoussi I, El Harti J, Ibrahimi A. Repurposing of known anti-virals as potential inhibitors for SARS-CoV-2 main protease using molecular docking analysis. *Bioinformat.* 2020;16(4):301-305. https://www.ncbi.nlm.nih.gov/pmc/articles/PMC7392094/pdf/97320630016301.pdf.

79. Van Damme E, De Meyer S, Bojkova D, Ciesek S, Cinatl J, De Jonghe S, et al. In vitro activity of itraconazole against SARS-CoV-2. *bioRxiv.* 2020:2020.11.13.381194. doi:10.1101/2020.11.13.381194.

80. Liu X, Wang XJ. Potential inhibitors against 2019-ncov coronavirus m protease from clinically approved medicines. *J Genet Genom.* 2020;47(2):119-121. doi:10.1016/j.jgg.2020.02.001

81. Jiang Y, Liu L, Manning M, Bonahoom M, Lotvola A, Yang Z, et al. Structural analysis, virtual screening and molecular simulation to identify potential inhibitors targeting 2'-O-ribose methyltransferase of SARS-CoV-2 coronavirus. *J Biomol Struct Dyn.* 2020:1-16. doi:10.1080/07391102.2020.1828172

82. Jiang Y, Liu L, Manning M, Bonahoom M, Lotvola A, Yang Z-Q. Repurposing therapeutics to identify novel inhibitors targeting 2'-o-ribose methyltransferase nsp16 of SARS-CoV-2. *ChemRxiv.* 2020:chemrxiv.12252965.v1. doi:10.26434/chemrxiv.12252965.v1.

83. Wu C, Liu Y, Yang Y, Zhang P, Zhong W, Wang Y, et al. Analysis of therapeutic targets for SARS-CoV-2 and discovery of potential drugs by computational methods. *Acta Pharm Sin B.* 2020;10(5):766-788. doi:10.1016/j.apsb.2020.02.008

84. Ghahremanpour MM, Tirado-Rives J, Deshmukh M, Ippolito JA, Zhang CH, Cabeza de Vaca I, et al. Identification of 14 known drugs as inhibitors of the main protease of SARS-CoV-2. *ACS Med Chem Lett.* 2020;11(12):2526-2533. doi:10.1021/acsmedchemlett.0c00521

85. Trezza A, Iovinelli D, Santucci A, Prischi F, Spiga O. An integrated drug repurposing strategy for the rapid identification of potential SARS-CoV-2 viral inhibitors. *npj Sci Rep.* 2020;10(1):13866. doi:10.1038/s41598-020-70863-9

86. da Silva FMA, da Silva KPA, de Oliveira LPM, Costa EV, Koolen HH, Pinheiro MLB, et al. Flavonoid glycosides and their putative human metabolites as potential inhibitors of the SARS-CoV-2 main protease (Mpro) and RNA-dependent RNA polymerase (RdRp). *Mem Inst Oswaldo Cruz.* 2020; 115:e200207. doi: 10.1590/0074-02760200207.

87. Maffucci I, Contini A. In silico drug repurposing for SARS-CoV-2 main proteinase and spike proteins. *J Proteome Res.* 2020;19(11):4637-4648. doi:10.1021/acs.jproteome.0c00383

88. Bank S, Basak N, Girish G, De SK, Maiti S. In-silico analysis of potential interaction of drugs and the SARS-CoV-2 spike protein. *Res Square.* 2020:rs.3.rs-30401/v1. doi:10.21203/rs.3.rs-30401/v1.

89. Shankar U, Jain N, Majee P, Mishra SK, Rathi B, Kumar A. Potential drugs targeting nsp16 protein may corroborates a promising approach to combat SARS-CoV-2 virus. *ChemRxiv.* 2020:chemrxiv.12279671.v1. doi:10.26434/chemrxiv.12279671.v1.

90. Peterson L. COVID-19 and flavonoids: In silico molecular dynamics docking to the active catalytic site of SARS-CoV and SARS-CoV-2 main protease. *SSRN.* 2020:3599426. doi:http://dx.doi.org/10.2139/ssrn.3599426.

91. Senathilake K, Samarakoon S, Tennekoon K. Virtual screening of inhibitors against spike glycoprotein of SARS-CoV-2: A drug repurposing approach. *Preprints.* 2020:202003.0042.v2. doi:10.20944/preprints202003.0042.v2.

92. Galvez J, Zanni R, Galvez-Llompart M. Drugs repurposing for coronavirus treatment: Computational study based on molecular topology. *Nereis.* 2020(12):15-18.

93. Chtita S, Belhassan A, Aouidate A, Belaidi S, Bouachrine M, Lakhlifi T. Discovery of potent SARS-CoV-2 inhibitors from approved antiviral drugs via docking and virtual screening. *Comb Chem High Throughp Scr.* 2021;24(3):441-454. doi:10.2174/1386207323999200730205447

94. Oliveira MD, Oliveira KM. Comparative docking of SARS-CoV-2 receptors antagonists from repurposing drugs. *ChemRxiv.* 2020:chemrxiv.12044538.v4. doi:10.26434/chemrxiv.12044538.v4.

95. Alves VM, Bobrowski T, Melo-Filho CC, Korn D, Auerbach S, Schmitt C, et al. QSAR modeling of SARS-CoV M(pro) inhibitors identifies sufugolix, cenicriviroc, proglumetacin, and other drugs as candidates for repurposing against SARS-CoV-2. *Mol Inform.* 2021;40(1):e2000113. doi:10.1002/minf.202000113

96. Wang J. Fast identification of possible drug treatment of coronavirus disease-19 (COVID-19) through computational drug repurposing study. *J Chem Inf Model.* 2020;60(6):3277-3286. doi:10.1021/acs.jcim.0c00179

97. Beck BR, Shin B, Choi Y, Park S, Kang K. Predicting commercially available antiviral drugs that may act on the novel coronavirus (SARS-CoV-2) through a drug-target interaction deep learning model. *Comp Struct Biotechnol J.* 2020;18:784-790. doi:10.1016/j.csbj.2020.03.025

98. White MA, Lin W, Cheng X. Discovery of COVID-19 inhibitors targeting the SARS-CoV-2 nsp13 helicase. *J Phys Chem Lett.* 2020;11(21):9144-9151. doi:10.1021/acs.jpclett.0c02421

99. Ekins S, Mottin M, Ramos P, Sousa BKP, Neves BJ, Foil DH, et al. Deja vu: Stimulating open drug discovery for SARS-CoV-2. *Drug Discov Today.* 2020;25(5):928-941. doi:10.1016/j.drudis.2020.03.019

100. Qiao Z, Zhang H, Ji HF, Chen Q. Computational view toward the inhibition of SARS-CoV-2 spike glycoprotein and the 3cl protease. *Computat.* 2020;8(2):53. doi:10.3390/computation8020053

101. Matsuyama S, Kawase M, Nao N, Shirato K, Ujike M, Kamitani W, et al. The inhaled steroid ciclesonide blocks SARS-CoV-2 RNA replication by targeting the viral replication-transcription complex in cultured cells. *J Virol.* 2020;95(1):JVI.01648-01620. doi:10.1128/JVI.01648-20

102. Nakajima K, Ogawa F, Sakai K, Uchiyama M, Oyama Y, Kato H, et al. A case of coronavirus disease 2019 treated with ciclesonide. *Mayo Clin Proc.* 2020;95(6):1296-1297. doi:10.1016/j.mayocp.2020.04.007

103. Zhou G, Stewart L, Reggiano G, DiMaio F. Computational drug repurposing studies on SARS-CoV-2 protein targets. *ChemRxiv.* 2020:chemrxiv.12315437.v1. doi:10.26434/chemrxiv.12315437.v1.

**Table S2**. Binding interactions with RdRP binding site for top 10 ranked drugs.

| No | Drug | Interacting residues |
| --- | --- | --- |
| 1 | Beclabuvir | Arg553, Lys621, Asp618, Tyr619, Pro620, Asp623, Arg624, Ser759, Asp760, Asp761, Lys798, Glu811, Phe812, Ser814, |
| 2 | Bemcentinib | Tyr455, Arg553, Lys621, Cys622, Asp623, Asp760, Asp761, Lys798, Glu811, Ser814 |
| 3 | Digoxin | Tyr455, Arg553, Trp617, Asp618, Lys621, Cys622, Asp623, Arg624, Asp760, Asp761, Trp800, Glu811, Phe812, Cys813, Ser814. |
| 4 | Galidesvir | Asp618, Tyr619, Asp760, Asp761, Trp800, Glu811, Phe812, Ser814 |
| 5 | Ivermectin | Arg553, Arg555, Trp617, Asp618, Asp623, Ser682, Thr687, Asp760, Asp761, Glu811, Cys813, Ser814 |
| 6 | Paritaprivir | Tyr455, Arg553, Asp618, Tyr619, Pro620, Lys621, Asp623, Arg624, Asp760, Asp761, Lys798, Ser814 |
| 7 | Remdesivir | Tyr455, Arg553, Tyr619, Trp617, Lys621, Asp623, Cys622, Arg624, Asp760, Asp761, Lys798, Trp800, Glu811 |
| 8 | Setrobuvir | Arg553, Trp617, Asp618, Lys621, Pro620, Asp623, Asp760, Asp761, Glu811 |
| 9 | Silibinin | Arg553, Trp617, Asp618, Tyr619, Asp623, Thr680, Thr687, Asp760, Asp761 |
| 10 | Voxilaprevir | Lys551, Arg553, Arg555, Asp618, Tyr619, Lys621, Asp623, Asp760, Asp761, Lys798, Glu811, Cys813, Ser814 |

**Table S3**. Top 20 natural drugs predicted to bind and inhibit RdRP. The drug descriptions are taken directly from DrugBank.

| Drug | Structure | Mode of Action | Binding Energy (kcal/mol) |
| --- | --- | --- | --- |
| Ivermectin | 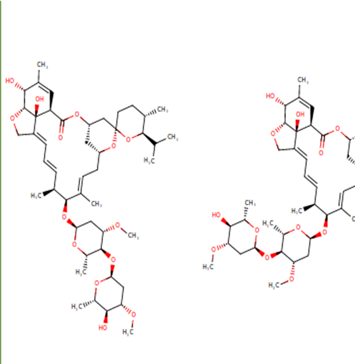 | Ivermectin is a broad-spectrum anti-parasite medication. Ivermectin is mainly used in humans in the treatment of onchocerciasis but is also effective against other worm infestations (such as strongyloidiasis, ascariasis, trichuriasis and enterobiasis). | -69.65 |
| Digoxin | 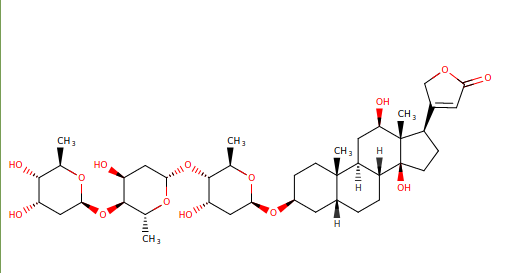 | This drug originates from the foxglove plant, also known as the Digitalis plant. Digoxin is one of the oldest cardiovascular medications used today. It is a common agent used to manage atrial fibrillation and the symptoms of heart failure | -54.35 |
| Silibinin | 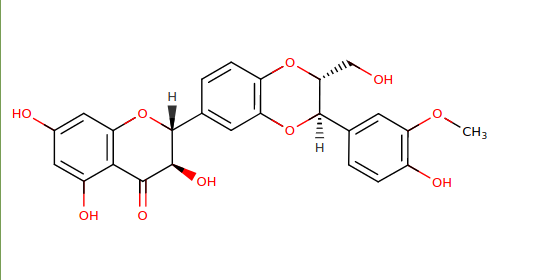 | Silibinin is the major active constituent of silymarin, a standardized extract of the milk thistle seeds, containing a mixture of flavonolignans consisting of silibinin, isosilibinin, silicristin, silidianin and others. in vitro and animal research suggest that silibinin has hepatoprotective (antihepatotoxic) properties that protect liver cells against toxins. | -57.23 |
| Rapamycin | 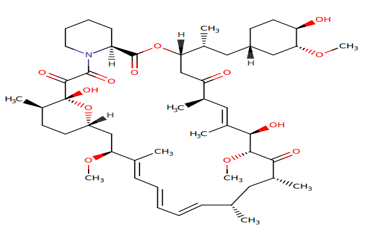 | A macrolide compound obtained from Streptomyces hygroscopicus that acts by selectively blocking the transcriptional activation of cytokines thereby inhibiting cytokine production. It is a potent immunosuppressant and possesses both antifungal and antineoplastic properties. | -45.20 |
| Novobiocin | 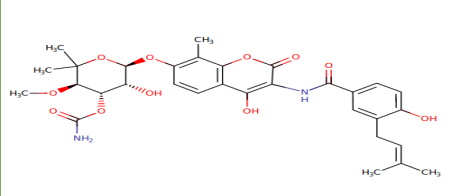 | Novobiocin is an antibiotic compound derived from Streptomyces niveus. Novobiocin binds to DNA gyrase, and blocks adenosine triphosphatase (ATPase) activity. | -45.25 |
| Ergotamine | 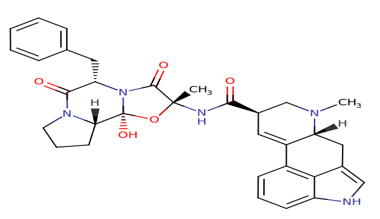 | A vasoconstrictor found in ergot of Central Europe. It is an alpha-1 selective adrenergic agonist and is commonly used in the treatment of migraine disorders. | -45.55 |
| Hesperidin | 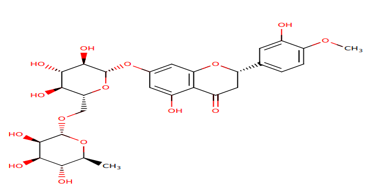 | Hesperidin is a flavan-on glycoside found in citrus fruits. | -45.87 |
| Ouabain | 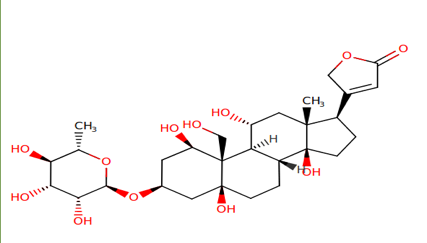 | A cardioactive glycoside consisting of rhamnose and ouabagenin, obtained from the seeds of Strophanthus gratus and other plants of the Apocynaceae. | -38.52 |
| Rutin | 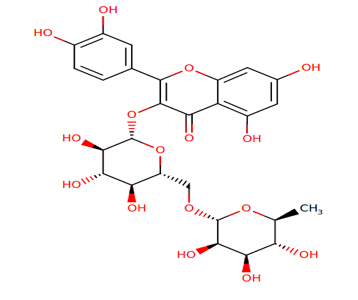 | A flavonol glycoside found in many plants, including buckwheat; tobacco; forsythia; hydrangea; viola, etc. It has been used therapeutically to decrease capillary fragility | -30.21 |

**Figure S1**. LigPlot (left) and hydrophobic protein surface representation (right) of the main interactions between RdRP and ergotamine.


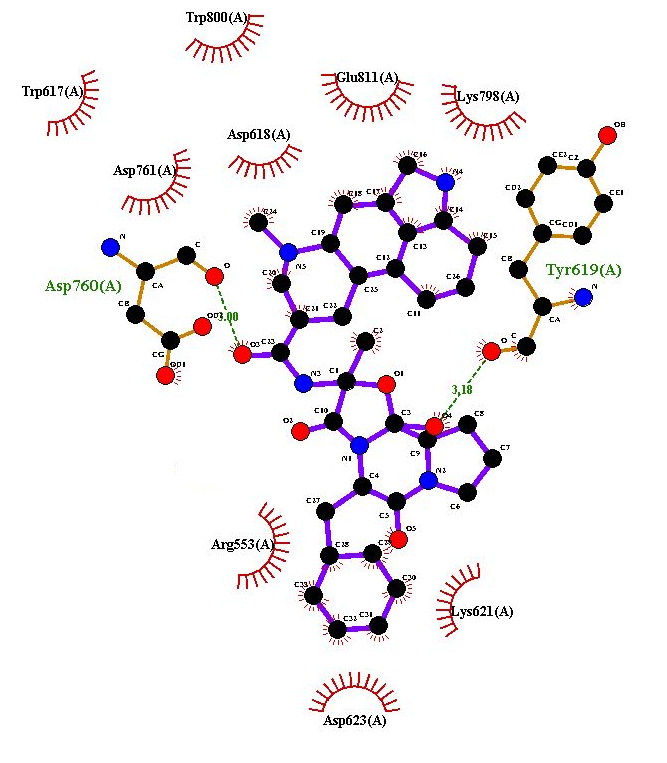

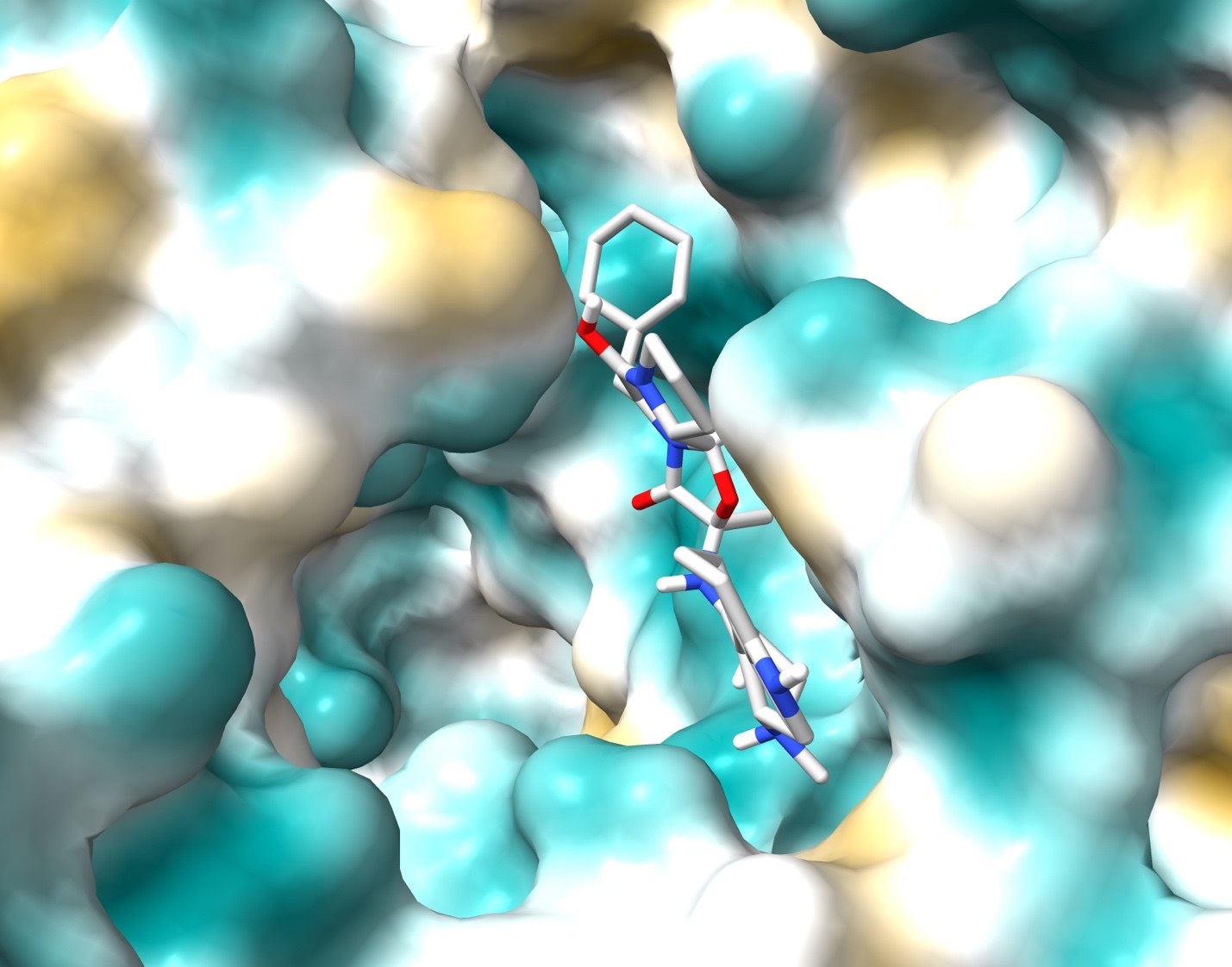


**Figure S2**. LigPlot (left) and hydrophobic protein surface representation (right) of the main interactions between RdRP and bemcentinib.


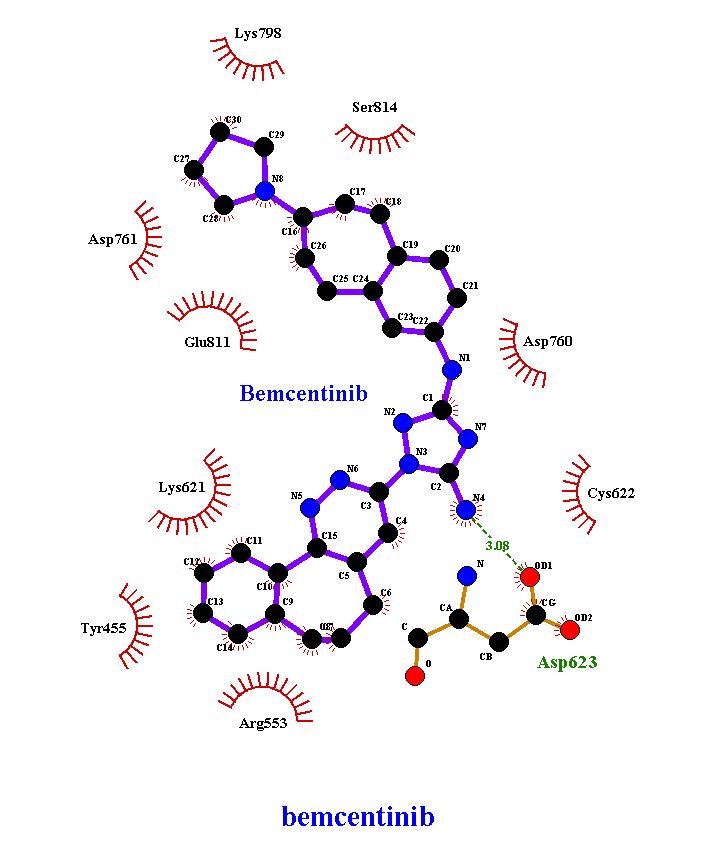

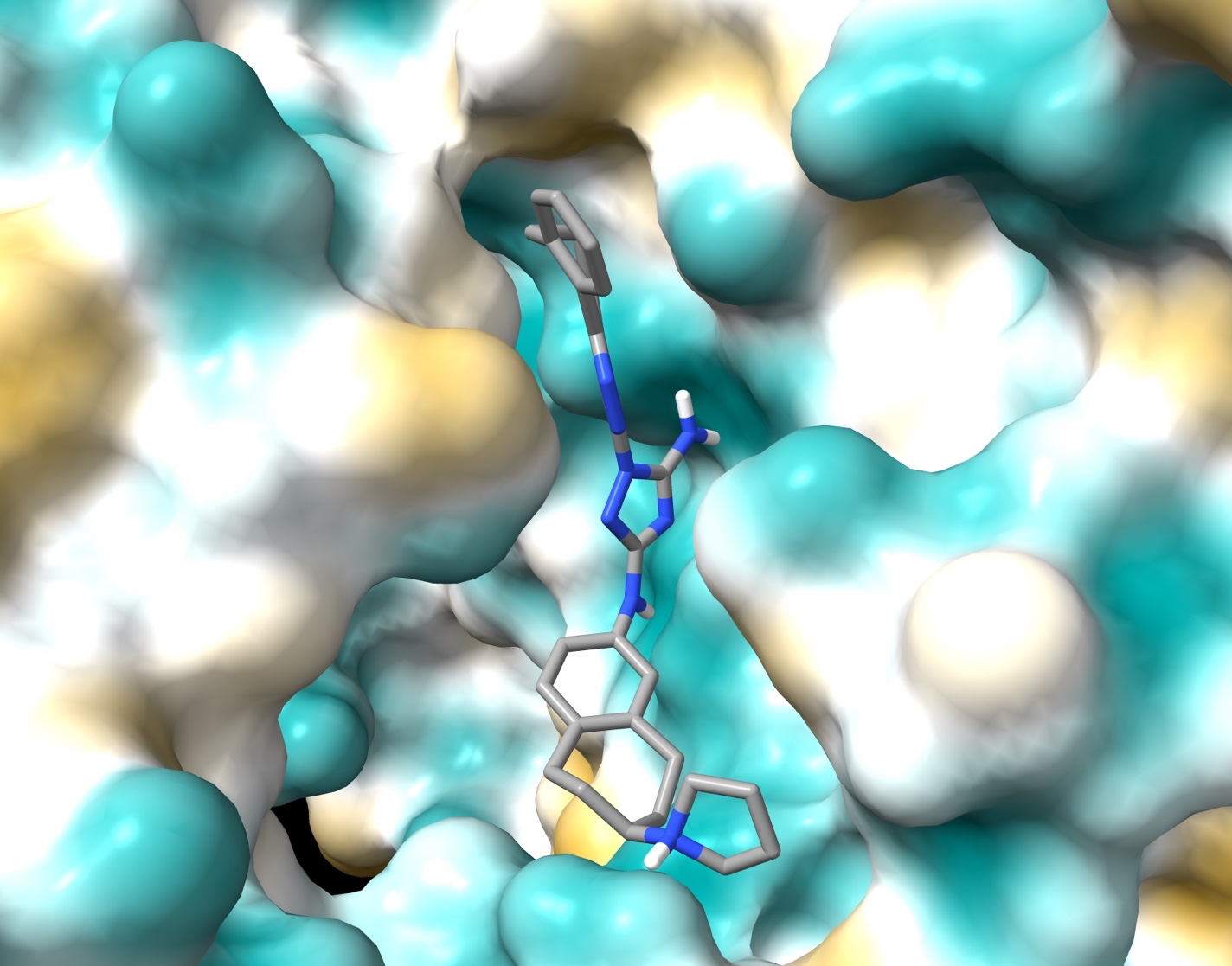


**Scripts Used**:

**1)Conf.txt**

receptor = 6Y2F.pdbqt

center_x= 9.245

center_y= -0.788

center_z = 18.371

size_x = 50

size_y = 50

size_z = 50

num_modes = 10

exhaustiveness = 50

**2)vina_screen.sh**

#! /bin/bash

for f in CHEMBL*.pdbqt; do

b=`basename $f .pdbqt`

echo Processing ligand $b

mkdir -p $b

vina --config conf.txt --cpu 50 --ligand $f --out $[b]/out.pdbqt --log $[b]/log.txt

done

**3)Script1.py**

#! /usr/bin/env python

import sys

import glob

def doit(n):

file_names = glob.glob('*/*.pdbqt')

everything = []

failures = []

print 'Found', len(file_names), 'pdbqt files'

for file_name in file_names:

file = open(file_name)

lines = file.readlines()

file.close()

try:

line = lines[1]

result = float(line.split(':')[1].split()[0])

everything.append([result, file_name])

except:

failures.append(file_name)

everything.sort(lambda x,y: cmp(x[0], y[0]))

part = everything[:n]

for p in part:

print p[1],

print

if len(failures) > 0:

print 'WARNING:', len(failures), 'pdbqt files could not be processed'

if __name__ == '__main__':

doit(int(sys.argv[1]))

**Supplementary References**
